# Limiting replication stress during somatic cell reprogramming reduces genomic instability in induced pluripotent stem cells

**DOI:** 10.1038/ncomms9036

**Published:** 2015-08-21

**Authors:** Sergio Ruiz, Andres J. Lopez-Contreras, Mathieu Gabut, Rosa M. Marion, Paula Gutierrez-Martinez, Sabela Bua, Oscar Ramirez, Iñigo Olalde, Sara Rodrigo-Perez, Han Li, Tomas Marques-Bonet, Manuel Serrano, Maria A. Blasco, Nizar N. Batada, Oscar Fernandez-Capetillo

**Affiliations:** 1 Genomic Instability Group, Spanish National Cancer Research Centre, 3, Melchor Fernandez Almagro, 28029 Madrid, Spain; 2 Ontario Institute for Cancer Research, Toronto, Ontario, Canada M5G 0A3; 3 Department of Medical Biophysics, University of Toronto, Toronto, Ontario, Canada M5G 1L7; 4 Telomeres and Telomerase Group, Spanish National Cancer Research Centre, 28029 Madrid, Spain; 5 Cellular Plasticity and Disease Modelling group, Department of Developmental and Stem Cell Biology, Institut Pasteur, 75015 Paris, France; 6 ICREA at the Institut de Biologia Evolutiva (Universitat Pompeu Fabra/CSIC), 08003 Barcelona, Spain; 7 Centro Nacional de Análisis Genómico (CNAG), 08028 Barcelona, Spain; 8 Tumor Supression Group, Spanish National Cancer Research Centre, 28029 Madrid, Spain

## Abstract

The generation of induced pluripotent stem cells (iPSC) from adult somatic cells is one of the most remarkable discoveries in recent decades. However, several works have reported evidence of genomic instability in iPSC, raising concerns on their biomedical use. The reasons behind the genomic instability observed in iPSC remain mostly unknown. Here we show that, similar to the phenomenon of oncogene-induced replication stress, the expression of reprogramming factors induces replication stress. Increasing the levels of the checkpoint kinase 1 (CHK1) reduces reprogramming-induced replication stress and increases the efficiency of iPSC generation. Similarly, nucleoside supplementation during reprogramming reduces the load of DNA damage and genomic rearrangements on iPSC. Our data reveal that lowering replication stress during reprogramming, genetically or chemically, provides a simple strategy to reduce genomic instability on mouse and human iPSC.

## Abstract (web summary)

The expression of reprogramming factors can induce replication stress in induced pluripotent stem cells. In this study, to reduce such genomic instability, Ruiz *et al.* increase CHK1 kinase levels and nucleoside supplementation during reprogramming.

## Graphical abstract

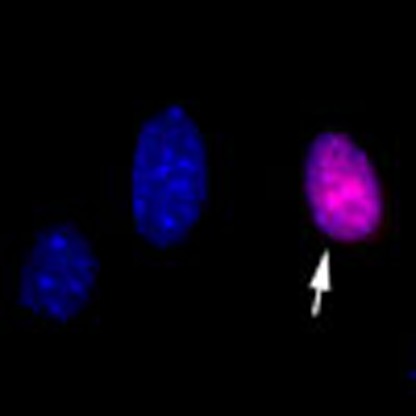

Reprogramming of somatic cells into induce pluripotent stem cells (iPSC) can be achieved by the expression of defined sets of transcription factors^1^ (for example, OCT4, SOX2, KLF4 and cMYC; OSKM hereafter). However, recent reports have shown evidence of DNA damage and genomic instability in iPSC^2^,^3^,^4^,^5^,^6^,^7^,^8^, raising concerns on their potential biomedical use. The source of genomic instability on iPSC remains unresolved, although several evidence suggest that it could be linked to replication stress (RS), a type of DNA damage occurring at stalled replication forks and limited by the ataxia telangiectasia and Rad3-related (ATR) and checkpoint kinase 1 (CHK1) kinases^9^. While the causes of RS are still not fully understood, some of the sources include insufficient levels of deoxynucleotides^10^, reduced levels of replication factors^11^, or mutations in DNA repair and replication factors (reviewed in ref. 9).

According to the oncogene-induced DNA damage model of cancer development, the expression of oncogenes leads to genomic instability in cancer cells through the generation of RS^12^. Interestingly, and besides the well-established role of cMYC, the remaining three factors of the OSKM set have also been shown to play oncogenic roles^13^,^14^,^15^. Hence, we hypothesized that similar to oncogene-induced RS; an analogous reprogramming-induced RS could drive genomic instability in iPSC. Supporting this view, we and others have recently demonstrated that iPSC contain genomic structural variations such as copy number variants (CNV) that were highly enriched in fragile sites^3^,^7^,^8^, a hallmark of RS. Furthermore, mouse embryonic fibroblasts (MEF) with reduced levels of ATR and which are highly sensitive to RS and resistant to transformation by oncogenes^16^,^17^, are also refractory to reprogramming (our own observations). In this work, we provide evidence for RS occurring at the reprogramming process and to understand the mechanisms underlying this RS. If RS were to significantly contribute to the genomic rearrangements found in iPSC, we reasoned that strategies directed to lowering reprogramming-induced RS could offer a strategy to reduce genomic instability on iPSC.

## Results

### The expression of reprogramming factors generates RS

**Figure 1:**
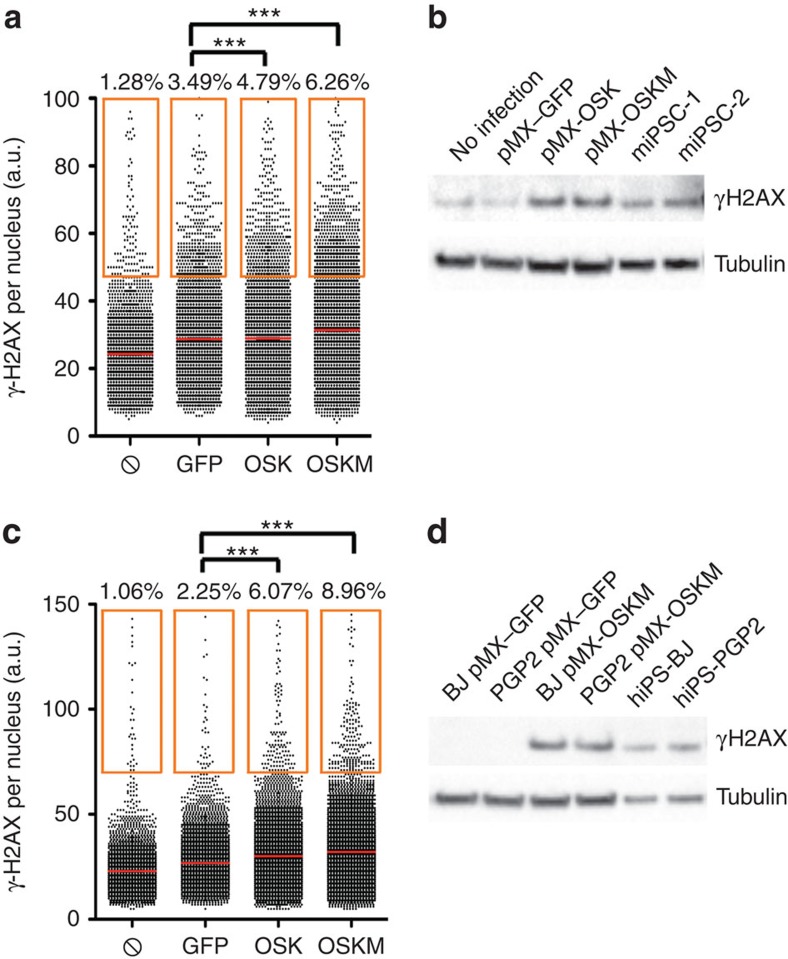
Expression of reprogramming factors generates replication stress. (**a**,**c**) HTM-mediated quantification of γH2AX intensity levels in MEFs (**a**) or BJ human fibroblasts (**c**) 4 days after infection with retroviruses encoding GFP, OSK or OSKM. Uninfected cells (struck through circle) were also included as negative control. Centre lines indicate mean values, whereas squared boxes show the percentage of outliers. Data are representative of three independent experiments performed in triplicate ***P<0.001. (**b**,**d**) Western blot analyses of cell extracts obtained from MEFs (**b**) or BJ human fibroblasts (**d**) 4 days after infection with retroviruses encoding GFP, OSK or OSKM. Uninfected cells (struck through circle), human (BJ or PGP2-derived iPSC lines) or mouse iPSCs were also included as controls. See Supplementary Fig. 14 for full western blot images.

First, we evaluated to what extent DNA damage occurred during reprogramming by analysing the levels of H2AX phosphorylation (γH2AX). High-throughput microscopy (HTM) and western blot analyses revealed increased levels of γH2AX in MEF (Fig. 1a,b; Supplementary Fig. 1) and human fibroblasts (Fig. 1c,d; Supplementary Fig. 2) expressing OSK, when compared with green fluorescent protein (GFP)-expressing cells or uninfected control cells. Furthermore, these levels were further augmented in the presence of cMYC. To discard the impact of viral integration, which could cause DNA breaks and induce γH2AX, we used a previously reported fibroblast-like human cell line, which expresses OSK in response to doxycycline^18^ (dFib-ind^OSK^) (Supplementary Fig. 3a). The expression of OSK in these cells induced γH2AX in a dose-dependent manner, which could again be further potentiated by the inclusion of cMYC (Supplementary Fig. 3b–e). Next, as direct measure of RS, we observed that replication fork speed, measured by single molecule DNA combing analysis, is lower in cells expressing OSKM than in GFP-expressing cells (Supplementary Fig. 3f). Interestingly, fork symmetry was not altered in OSKM-expressing fibroblasts when compared with GFP control cells (ratio of short to long tracks: GFP=0.7429±0.178, OSKM=0.7341±0.1867; *P*=0.96). Finally, since cells suffering from RS are particularly dependent on RS-response kinases^17^,^19^,^20^, we tested the effect of lowering ATR and CHK1 activity during reprogramming. Addition of ATR or CHK1 inhibitors interfered with the successful generation of iPSC colonies in a dose-dependent manner (Supplementary Fig. 4). Collectively, these data demonstrate that expression of reprogramming factors induces RS.

### Strategies to reduce reprogramming-induced RS

**Figure 2:**
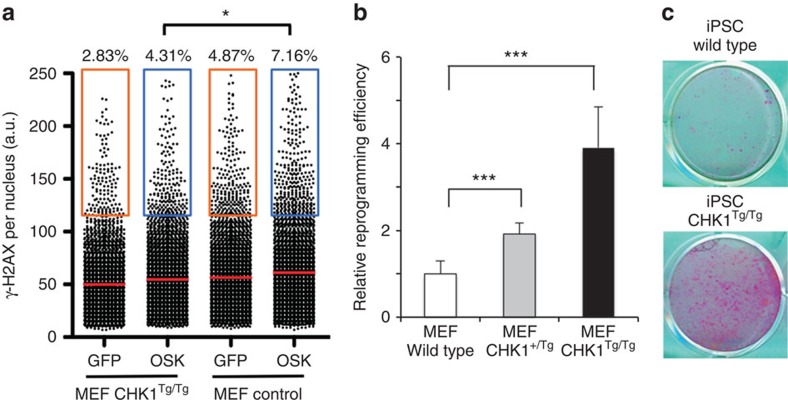
Increased CHK1 levels facilitate reprogramming and reduce RS on iPSC. (**a**) HTM-mediated quantification of γH2AX intensity levels in wild-type or *Chk1*^TG/TG^ MEFs 4 days after infection with retroviruses encoding GFP or OSK. Centre lines indicate mean values, whereas squared boxes show the percentage of outliers. Data are representative of two independent experiments performed in triplicate *P<0.05. (**b**) Wt, *Chk1*^TG/+^ or *Chk1*^TG/TG^ MEF were infected with retroviruses encoding OSK and relative reprogramming efficiencies were evaluated by counting alkaline-phosphatase-positive colonies. Data are representative of three independent experiments performed in triplicate in MEF derived from four different embryos. ***P<0.001. (**c**) Representative images of the analysis described in **b**.

RS-generating oncogenes frequently upregulate CHK1 as a way to limit transformation-associated RS^21^,^22^,^23^. Similarly, OSK expression in human fibroblasts increases CHK1 levels (Supplementary Fig. 5). Importantly, cMYC was excluded from the reprogramming cocktail since it transcriptionally induces CHK1 expression^21^. We recently reported the generation of mice selectively protected against RS, which harbour one additional allele of the *Chk1* gene^24^ (*Chk1*^TG^). In agreement with the idea that oncogenes upregulate CHK1 to limit RS^24^, *Chk1*^TG^ MEF present lower levels of oncogene-induced RS and transform more efficiently than wild-type (wt) cells with Ras and E1A oncogenes^24^. Similarly, *Chk1*^TG^ MEF presented lower levels of reprogramming-induced γH2AX (Fig. 2a). Thus, given our previous observations on oncogene-induced RS, we hypothesized that *Chk1*^TG^ MEF could reprogramme more efficiently than wt cells. In fact, we observed an increase in the reprogramming efficiency of *Chk1*^TG^ cells, which was more pronounced on *Chk1*^TG/TG^ than on *Chk1*^TG/+^ MEF (Fig. 2b,c; Supplementary Fig. 6). Consistent with MEF data, overexpression of CHK1 also reduced OSK-induced γH2AX levels in human fibroblasts (Supplementary Fig. 7a). In addition, increased CHK1 levels boosted reprogramming efficiencies on the inducible human dFib-ind^OSK^ system (Supplementary Fig. 7b). This effect was also noticeable even in the presence of high doses of doxycycline, which implies a higher expression of reprogramming factors, higher RS-driven toxicity and, as a consequence, lower reprogramming efficiencies. When cMYC was introduced in the cocktail, expression of CHK1 increased reprogramming efficiency specifically at high doses of doxycycline (Supplementary Fig. 7c). Altogether, these data reveal that reprogramming factors induce RS and upregulate CHK1 in an attempt to prevent replicative damage. In this context, increasing CHK1 levels confers an additional protection from reprogramming-induced RS and increases the efficiency of iPSC generation.

**Figure 3:**
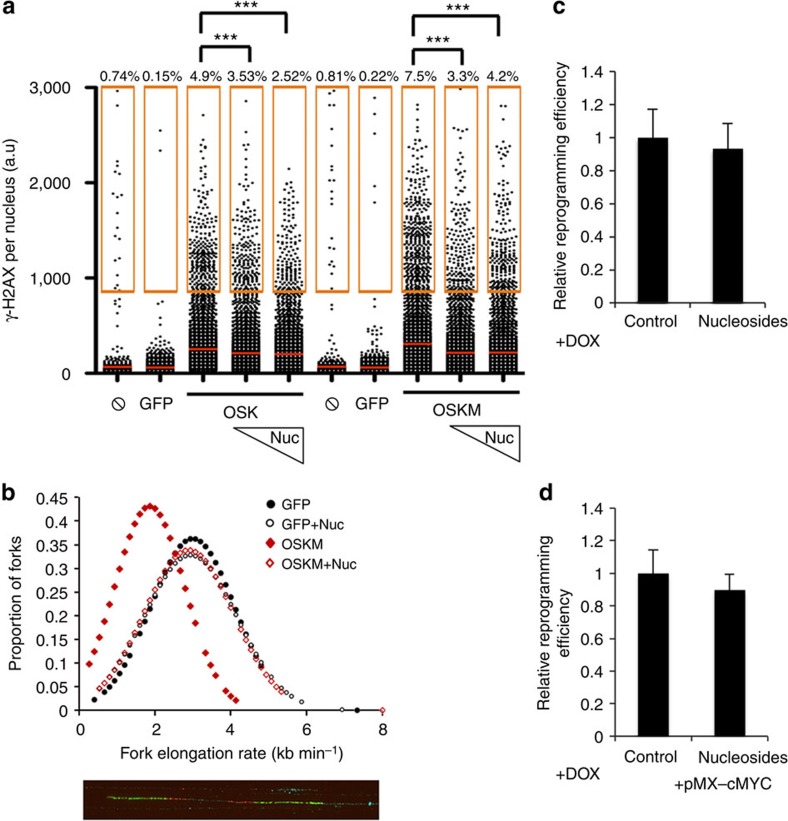
Nucleoside supplementation decreases RS in cells expressing reprogramming factors. (**a**) HTM-mediated quantification of γH2AX intensity levels in PGP2 human fibroblasts 4 days after infection with OSK or OSKM with or without daily addition of nucleosides. Centre lines indicate mean values, whereas squared boxes show the percentage of outliers. Uninfected cells (
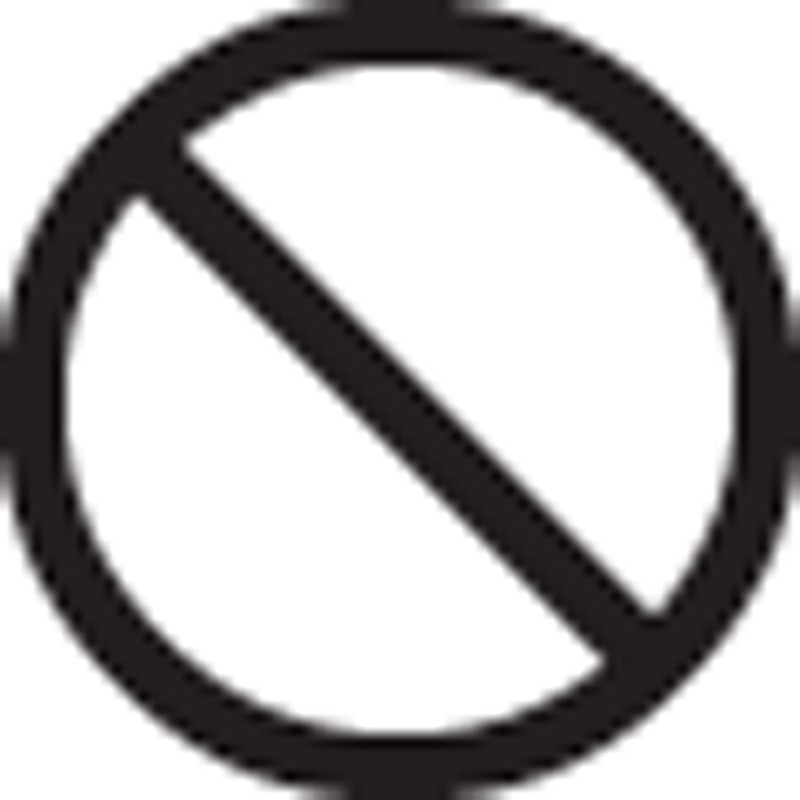
) were also included as negative control. Data are representative of three independent experiments performed in triplicate ***P<0.001. (**b**) Graphical representation of the fork elongation rate (kb min^−1^), measured by DNA combing, in BJ fibroblasts 4 days after infection with retroviruses encoding either GFP or OSKM with or without the addition of nucleosides. A representative image of a stained DNA fibre is shown in the lower part. Iododeoxyuridine (IdU, red) and Chlorodeoxyuridine (CIdU, green) signals are shown. (**c**,**d**) Relative reprogramming efficiency determined in RFP-infected (**c**) or RFP plus cMyc-infected (**d**) dFib-ind^OSK^ cells treated with doxycycline at a concentration of 100 ng ml^−1^ with or without daily addition of nucleosides. Reprogramming efficiency was evaluated by scoring the percentage of GFP-positive colonies (resulting from the reactivation of the endogenous OCT4 promoter) and RFP-negative colonies (resulting from the silencing of exogenous transgenes). Data are representative of two independent experiments performed in duplicate.

We next explored whether we could reduce reprogramming-induced RS by chemical means, without the need to add another vector to the reprogramming cocktail. While nucleotides are not cell-permeable, nucleoside supplementation has been shown to limit oncogene-induced RS^10^. Likewise, reprogramming-induced γH2AX levels were progressively lowered by increasing amounts of nucleosides (Fig. 3a; Supplementary Figs 8a and 9). Furthermore, this effect was independent of cMYC expression (Fig. 3a; Supplementary Figs 8b and 9). Moreover, nucleoside supplementation was also able to rescue the reduced fork speed observed after expression of reprogramming factors (Fig. 3b). Of note, and in contrast to CHK1, nucleosides suppressed reprogramming-induced RS but did not increase reprogramming efficiencies (Fig. 3c,d; Supplementary Fig. 8c,d). One possibility to explain the higher impact of CHK1 overexpression during reprogramming is that CHK1 not only suppresses RS but also arrests cells in G2, preventing the breakage of stalled forks during mitosis^25^. Overall, these data demonstrated the feasibility of decreasing the levels of reprogramming-induced RS by genetic or chemical means.

### Limiting RS reduces genomic instability in iPSC

**Figure 4:**
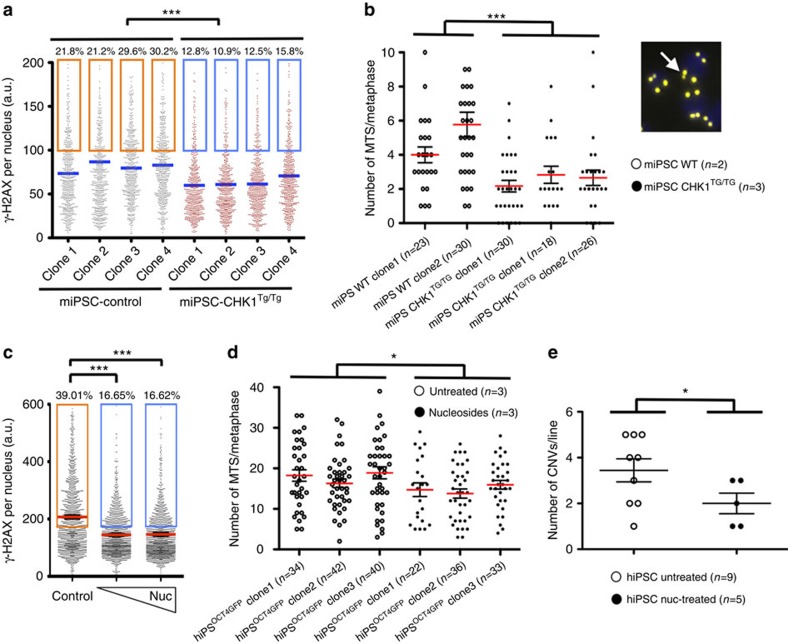
Lowering reprogramming-induced RS decreases genomic instability on iPSC. (**a**) HTM-mediated quantification of nuclear γH2AX intensity levels in four independent iPSC lines derived from wild-type or *Chk1*^TG/TG^ MEF. Centre lines indicate mean values, whereas squared boxes show the percentage of outliers. ***P<0.001. (**b**) Average number of multi-telomeric signal (MTS) per metaphase evaluated in iPSC lines derived from wild-type and *Chk1*^TG/TG^ MEF. Five different iPSC lines were used for quantification and at least 50 metaphases were evaluated per line. In the right, a representative image from the MTS analyses is shown. The white arrow indicates a chromosome with a fragmented multi-telomeric signal (MTS). (**c**) HTM-mediated quantification of γH2AX intensity levels in BJ-derived human iPSC lines generated with or without daily addition of different amounts of nucleosides. Centre lines indicate mean values, whereas squared boxes show the percentage of outliers. Data are representative of two independent experiments performed in triplicate ***P<0.001. (**d**) Average number of MTS per metaphase evaluated in iPSC lines derived from human dFib-ind^OSK^ cells untreated or treated with nucleosides during the whole reprogramming process. Three different iPSC lines per condition were used for quantification and at least 50 metaphases were evaluated per line. (**e**) SurePrint G3 Human High Resolution Microarrays were used to analyse the number of CNV in hiPSC clones untreated or treated with nucleosides during the whole reprogramming process. A one-tailed unpaired *t*-test was used to compare CNV data sets. *P<0.05.

We hypothesized that the RS that occurs during reprogramming might contribute to the genomic instability that has been observed in iPSC. Therefore, we reasoned that lowering reprogramming-induced RS could reduce genomic instability in induced pluripotent cells. To test this idea, we first analysed whether iPSC lines generated from *Chk1*^TG^ MEF presented lower levels of RS. Indeed, *Chk1*^TG^ iPSC lines presented lower amounts of γH2AX than their wt counterparts (Fig. 4a). In addition, we analysed the level of multi-telomeric signals (MTS), which derive from RS at telomeric regions, as a direct readout or RS-driven chromosomal fragility^26^,^27^. Again, the average number of MTS/metaphase was significantly lower in *Chk1*^TG^ iPSC lines compared with wt iPSC lines (Fig. 4b). Hence, the increased levels of CHK1 provided by an extra *Chk1* allele reduce RS and spontaneous chromosomal fragility on iPSC. Of note, iPSC lines derived from wt or *Chk1*^TG^ MEF were *bona fide* iPSC as they had silenced the expression of exogenous transgenes (Supplementary Fig. 1), expressed pluripotent markers at similar level to that observed in mouse embryonic stem cells (Supplementary Fig. 10a) and were able to participate in the formation of chimeric mice (Supplementary Fig. 10b).

Consistent with the data observed in MEF, human iPSC lines derived in the presence of exogenously added nucleosides presented lower amounts of γH2AX and a significantly lower number of MTS/metaphase compared with the levels observed in untreated iPSC (Fig. 4c,d). Finally, we reasoned that if reported CNVs found in iPSC derived from RS, nucleoside supplementation might limit the number of these genome rearrangements. We thus obtained several human iPSC lines generated in the presence or absence of nucleoside supplementation during the reprogramming process. DNA from hiPSC clones was used to evaluate the number of CNV by array comparative genomic hybridization (aCGH). Importantly, two independent reprogramming experiments showed that the average number of *de novo* CNVs was lower when reprogramming was done in the presence of nucleoside supplements (Fig. 4e; Supplementary Fig. 11; Supplementary Tables 1 and 2). It is noteworthy that most of the *de novo* CNV detected in the generated iPSC lines fall into either known fragile sites, sites of recurrent chromosomal rearrangements or loci that have been shown to undergo CNV in response to RS-generating agents such as the ribonucleotide reductase inhibitor hydroxyurea (HU) or the DNA polymerase inhibitor Aphidicolin^28^, providing strong support for the notion that the *de novo* CNV observed on iPSC are due to RS (Supplementary Tables 1 and 2). Specifically, 68% of the 41 CNV shown in Fig. 4e have been previously linked to RS (12 are known fragile sites and 16 are at sites that underwent HU and or Aphidicolin-induced CNV). The nucleoside supplementation conditions defined here did not affect the pluripotency of hiPSC, as determined by the expression of pluripotent markers and their capacity to silence exogenous transgenes (Supplementary Fig. 12). Furthermore, we verified that the nucleoside supplementation conditions that lower CNV numbers on iPSC are not mutagenic. To this end, we exome sequenced a total of five untreated and five nucleosides treated human iPSC lines and observed a similar number of point mutations in coding regions regardless of the treatment (Supplementary Table 3). The number of point mutations is similar to what had been previously reported for iPSC, which, as suggested, is likely a reflection of mutations harboured in some of the parental fibroblasts that expanded during reprogramming^2^,^4^,^29^,^30^,^31^. While not definitive, these data are consistent with the rest of the evidences provided in this manuscript, which reveal that lowering RS during reprogramming reduces genomic instability in the resultant iPSC.

## Discussion

The findings of genomic instability in iPSC casts doubts on their safety, due to the potential consequences of these mutations. Thus, it is necessary to define culture conditions or protocols for the generation of iPSC that can reduce genomic instability on the reprogrammed cells. Here we show, in human and mouse cells, that reprogramming is associated with increased levels of RS, which, at least partially, contribute to the genomic instability of iPSC. Furthermore, we demonstrate that reducing the levels of RS during the reprogramming process limits the amount of genomic aberrations and spontaneous chromosomal fragility found on reprogrammed cells.

Several lines of evidence support our finding that RS is taking place during reprogramming. First, we observed elevated levels of pan-nuclear γH2AX in cells expressing the reprogramming factors, which we and others have shown in the past is a hallmark of cells enduring RS^16^,^17^,^20^,^32^,^33^. Second, DNA replication fork speed is lowered by the expression of the reprogramming factors. Interestingly, the reduced fork speed that arises during reprogramming occurs in the absence of fork asymmetry. One possibility to explain this observation is that reprogramming-induced RS could arise from insufficient pools of deoxynucleotides, which would lead to reduced fork speed but symmetrical forks. Accordingly, treatment of human cells with the ribonucleotide reductase inhibitor HU reduces fork speed but does not alter fork symmetry (Supplementary Fig. 13). This would be consistent with the rescue of RS that we see on nucleoside supplementation, or with the fact that a large fraction of the CNVs that we have detected on iPSC were previously reported as induced by HU^28^. If reprogramming factors directly alter the expression of enzymes involved in nucleotide biosynthesis, as previously shown for oncogenes^10^, remains to be seen. Finally, functional inhibition of the ATR/CHK1 kinases, critical effectors of the RS-response, interferes with cell reprogramming. In summary, and similar to oncogene-induced RS, our data support that a reprogramming-induced RS contributes to genomic instability in iPSC.

The molecular mechanisms behind reprogramming-induced RS remain obscure, although they would likely be similar to those proposed in other contexts where RS occurs. For instance, interference between DNA replication and transcription machineries, either by the physical presence of reprogramming factors or by the accumulation of RNA/DNA hybrids due to the enhanced expression levels, has been shown to drive RS and genomic rearrangements^34^. Alternatively, or in addition to, limiting amounts of nucleotides might not meet the high proliferation rates that are required for successful reprogramming^35^. This later hypothesis would be consistent with the high percentage of CNV observed on iPSC that were previously discovered as HU-induced CNV^28^. The observation that replication rates per fork are slowed down during reprogramming, but fork symmetry is not altered, further supports this view.

Our work not only documents the existence of reprogramming-induced RS, but also provides examples of strategies that can be used to lower this phenomenon: increasing CHK1 levels or the addition of nucleoside supplements to the culture media. Both approaches reduce significantly the level of DNA damage observed in iPSC. However, the addition of nucleosides, from a practical point of view, seems an easy procedure to implement. This simple practice to reduce genomic instability in iPSC was assessed by γH2AX levels, telomere fragility and the number of *de novo* CNV. Moreover, the conditions defined here did not increase somatic point mutations. Previous studies have reported the presence of CNV on pluripotent cells (either ESC or iPSC) maintained during prolonged time in culture^3^,^7^,^36^. We speculate that nucleoside supplementation might also serve to diminish the severity of these effects. Along these lines, it is interesting to note that the supplement we have used in this manuscript is a nucleoside solution that is commercialized for the growth of ESC cultures. Of note, the conditions defined in this manuscript only partially reduce the genomic instability of iPSC, which can also derive from other known sources of DNA damage such as reactive oxygen species^5^. Nevertheless, these initial studies provide proof-of-concept for the design of cell culture practices that can diminish DNA damage during reprogramming. Future research in this area will probably further reduce this unwanted off-target effect of the reprogramming process.

Reports documenting the presence of genomic instability on iPSC have raised concerns regarding their clinical use. Our work here illustrates that reprogramming-induced RS contributes to the genomic instability of iPSC, and reveals that the addition of nucleosides to the reprogramming medium provides a simple approach to reduce the number of genomic rearrangements that are generated during the reprogramming of somatic cells.

## Methods

### Cell culture

dFib-ind^OSK^ fibroblasts-like cells^18^, 293T cells (ATCC, CRL-3216), PGP2 (gift from George Church, Harvard University) and BJ human fibroblasts (ATCC, CRL-2522) were grown in DMEM (Invitrogen), 10%FBS and 0.1 mM non-essential amino acids. MEFs were obtained from 13.5 embryos by standard methods and cultured in DMEM, 15%FBS and 0.1 mM non-essential amino acids in low-oxygen conditions. The hESC line H9 (WA09), obtained from WiCell Research Institute, as well as the different hiPSC lines generated were cultured in Geltrex (Invitrogen) with mTeSR1 media (Stem Cell Technologies). miPSC clones were cultured on a feeder layer of inactivated MEFs with DMEM (high glucose) supplemented with 15% knockout serum replacement (Invitrogen), LIF (1,000 U ml^−1^), 0.1 mM non-essential amino acids, 1% glutamax and 55 μM β-mercaptoethanol.

### Plasmid construction

For the construction of pMX-CHK1, the coding sequences of *Chk1* were amplified by PCR from human complementary DNA. PCR products were digested with EcoRI and sub-cloned into an EcoRI-linearized pMX retroviral vector. The final construct was sequenced to rule out the presence of mutations.

### Retroviral production

Moloney-based retroviral pMX-derived vectors were co-transfected with packaging vectors in 293T cells using Lipofectamine 2000 (Invitrogen) to generate viral particles as previously described^18^.

### Human and mouse iPSC generation

Primary MEF (passage 2–4) were incubated with retroviral supernatants expressing the reprogramming factors up to four times (one infection every 12 h) after which the regular media was replaced by miPSC culture media (see above). Cultures were maintained until ES-cell-like colonies arose. Clones of ES-cell-like cells were then picked and expanded on feeder fibroblasts. To generate hiPSCs, BJ fibroblasts were infected with retroviral supernatants by infection of the cells at 1,850 r.p.m. for 1 h at room temperature in the presence of polybrene (4 μg ml^−1^). After two viral infections, cells were trypsinized and plated on top of irradiated MEFs. One day later, cells were switched to a media containing DMEM/F12 (Invitrogen), 20% knockout serum replacement, 0.1 mM non-essential amino acids, 1% glutamax, 55 μM β-mercaptoethanol and 10 ng ml^−1^ bFGF (Mylteni Biotec). In the case of dFib-ind^OSK^ fibroblasts-like cells, cells were also infected with the corresponding retroviral supernatants and the media was supplemented with doxycycline to induce the expression of OSK and promote reprogramming as described^18^. Where indicated, reprogramming cells were incubated with nucleosides (Embryomax ES-cell-qualified nucleosides, 100 × solution, Millipore), which were added daily. To evaluate reprogramming efficiencies, we plated the same number of infected BJ fibroblasts or dFib-ind^OSK^ fibroblasts-like cells on irradiated MEFs and the relative percentage of Nanog^+^ colonies compared with the colonies observed in the control was scored. Nanog detection was performed as described^18^. Reprogramming efficiency was additionally scored by evaluating the relative percentage of GFP+ (resulting from the endogenous reactivation of the OCT4 promoter) and RFP^−^(resulting from silencing of exogenous retroviral sequences encoding for red fluorescent protein (RFP)) colonies obtained from dFib-ind^OSK^-reprogrammed cells.

### RNA isolation and real-time PCR

Total RNA was isolated using the Absolutely RNA Microprep Kit (Stratagene) according to manufacturer's recommendations. Complementary DNA was synthesized using the SuperScript II Reverse Transcriptase kit for RT–PCR (Invitrogen). Real-time PCR was performed using the SYBR-Greener qPCR Supermix (Invitrogen) in the Cycler Real-Time PCR System (BioRad). GAPDH expression level was used to normalize values of gene expression. Data are shown as fold change relative to the sample control and at least two independent experiments in triplicate were performed. Sequences of the primers used in this work are shown in Supplementary Table 4.

### Western blot analysis

Cell pellets were lysed in 50 mM Tris pH 7.9/8 M urea/1%Chaps and incubated with shaking at 4 °C for at least 30 min. About 25 μg of supernatants were run and transferred for γH2AX detection by using a mouse monoclonal antibody against γH2AX (1:1,000, Upstate 05-636). Tubulin was used as control loading (1:10,000, Sigma).

### High-throughput microscopy

BJ fibroblasts, MEF, iPSC or dFib-ind^OSK^ fibroblast-like cells were plated on μCLEAR bottom 96-well plates (Greiner Bio-One). One day later, cells were untreated, infected by spinfection twice and/or incubated with nucleosides or doxycline as indicated. γH2AX staining was performed using standard procedures as described^24^ and images were automatically acquired in an Opera High-Content Screening System (Perkin Elmer). Each condition was performed in triplicate and at least 25 different fields per well at a × 20 magnification were taken. Images were segmented using a DAPI (4′,6-diamidino-2-phenylindole) staining to generate nuclear masks from which the mean γH2AX signal was calculated.

### Array comparative genomic hybridization

For the detection of CNV, genomic DNA isolated from iPSC and the original human fibroblasts was hybridized in SurePrint G3 Human High Resolution 1X1M Microarrays (CNV) (Agilent Technologies) following manufacturer's instructions. CNV regions in each sample were identified using a Reversible Jump Hidden Markov Model implemented in the software RJaCGH^37^ requiring an average posterior probability of the probes >0.75. Next, calls that did not fulfil the following criterion were filtered out: an average log2value of the region equal to the median±1.5 × s.d. of all log2values of the chip. CNVs were also identified using the aberration detection method (ADM-2: 6,5 value) algorithm suggested by Agilent Technologies. We established a log2 ratio (Cy5/Cy3) threshold of ±0,25 and the concurrence of at least four consecutive probes with differential signal intensity for consideration as a CNV. All iPSC lines were grown for equal number of days for a total of six passages before collecting samples for DNA isolation.

### DNA combing

Human BJ neonatal foreskin fibroblasts (5 × 10^5^) were infected with retroviruses encoding OSKM and media supplemented or not with nucleosides. After 72 h, cells were pulsed with IdU (100 μM) for 15 min at 37 C° and subsequently with CldU (100 μM) for 30 min at 37 C°. About 4.5 × 10^4^ cells were washed and resuspended in cold PBS and mixed in 1:1 ratio with PBS containing 1% low melting agarose, transferred to casting mould and allowed to solidify at 21 °C. Plugs were incubated in 0.5 ml of proteinase K buffer in a 12-ml round-bottom tube at 50 C° for 3 days, with buffer replaces after each 24-h period. Plugs were washed five times with 10 ml of TE at 21 °C and incubated in dark at 21 °C for 30 min in 100 μl of TE supplemented with 1.5 μl of YOYO-1. After four additional washes with TE, plugs were incubated with MES pH 5.7 for 20 min at 70 °C in the dark. About 3 U of beta-agarase (NEB) was added and further incubated overnight at 42 °C. DNA was combed onto silanized coverslip and subsequently heated for 2 h at 60 °C. Coverslips were dounced in 70, 90 and 100% EtOH in coplin jars, incubated for 25 min in 1 M NaOH and then incubated with anti-CldU (AbCys SA), anti-IdU (Becton Dickinson) and anti-ssDNA (Chemicon) in a humid chamber at 37 °C. Coverslips were washed five times with PBS/Tween and incubated with secondary antibodies (Molecular Probes) for 30 min at 37 °C. After washing with PBS, Prolong Gold Antifade mounting reagent was added to the coverslips and incubated at 21 °C for 2 h. Cells were imaged on Zeiss Observer microscope at × 63 objective. Fork asymmetry was defined as the ratio between the longest to the shortest track.

### Multi-telomeric signals

MTS were detected on metaphases after fluorescent *in situ* hybridization with a telomeric probe as previously described^38^. At least 50 metaphases per sample were scored for MTS by superimposing the telomere image on the DAPI chromosomes image.

### Exome sequencing

Enrichment of coding exons was done using Truseq exome capture following manufacturer's instructions. Libraries (Trueseq V2) were sequenced on a HiSeq sequencer with 101 × 2-paired end reads. Library generation, exome capture and sequencing for control and nucleoside-treated iPSC were carried out simultaneously.

### Identification of point mutations

Fastq files were aligned to the human reference (hg19) using Novoalign. Mapped reads were realigned, quality scores were recalibrated using GATK^39^ and PCR artifacts removed using Picard's MarkDuplicates (http://picard.sourceforge.net). Variants were called using GATK's unified genotyper^39^.

### Animal research

*Chk1* transgenic mice were kept under standard conditions at specific-pathogen-free facility of the Spanish National Cancer Centre in a mixed background^24^. All mouse work was performed in accordance with the Guidelines for Human Endpoints for Animals Used in Biomedical Research, and under the supervision of the Ethics Committee for Animal Research of the ‘Instituto de Salud Carlos III'.

## Additional information

**Accession codes:** The Exome sequencing data have been deposited in the Sequence Read Archive under accession number: SRP062342.

**How to cite this article:** Ruiz, S. *et al.* Limiting replication stress during somatic cell reprogramming reduces genomic instability in induced pluripotent stem cells. *Nat. Commun.* 6:8036 doi: 10.1038/ncomms9036 (2015).

## Supplementary Material

Supplementary Information Supplementary Figures 1-14, Supplementary Tables 1-4 and Supplementary References
