# Supplementary material for: Limiting replication stress during somatic cell reprogramming reduces genomic instability in induced pluripotent stem cells
Source: Nat Commun. 2015 Aug 21;6:8036. doi: 10.1038/ncomms9036 (PMC4560784; doi:10.1038/ncomms9036)
Supplement: Supplementary Information — Supplementary Figures 1-14, Supplementary Tables 1-4 and Supplementary References [file ncomms9036-s1.pdf]

Supplementary Figures:

Ruiz *et al* **Fig S1**

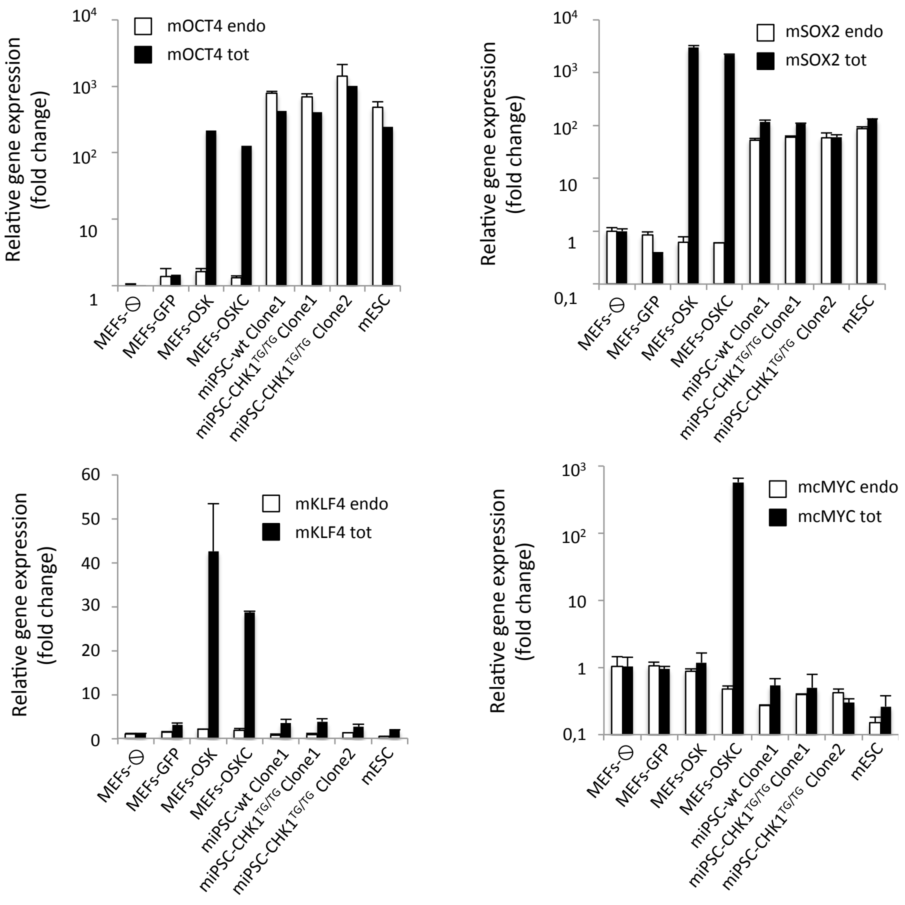

Supplementary Figure 1: Expression of reprogramming factors in mouse embryonic fibroblasts (MEF). Real-time PCR analysis to quantify relative expression levels of the indicated reprogramming factors four days after infection with retroviruses encoding GFP, OSK or OSKM. Two different sets of primers were used to examine the level of endogenous (endo) and total (tot: endogenous plus exogenous) expression for each factor in order to determine transgene-driven contribution. Different mouse iPSC lines and mESC were included to compare the levels of expression detected in pluripotent cells to retroviral-transduced cells. Note that miPSC showed a complete silencing of retroviral transgenes. Uninfected cells (⦸) were also included as negative control. Results are representative of two independent experiments performed in triplicate.

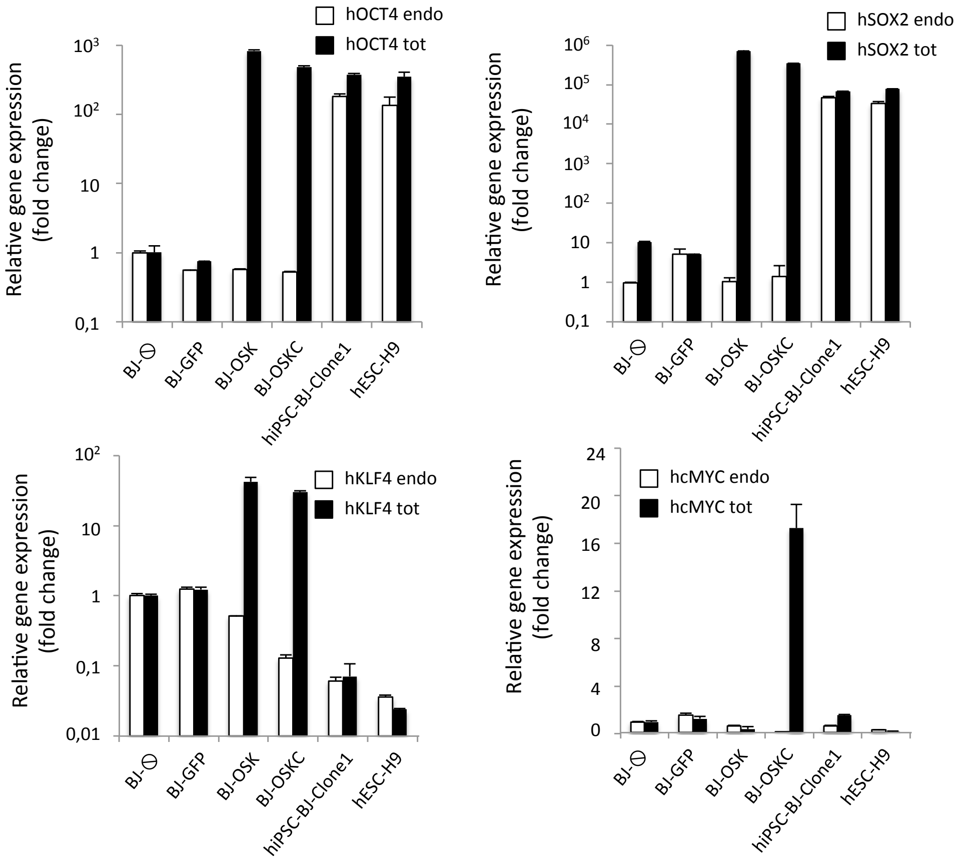

Supplementary Figure 2: Expression of reprogramming factors in BJ human fibroblasts. Real-time PCR analysis to quantify relative expression levels of the indicated reprogramming factors four days after infection with retroviruses encoding GFP, OSK or OSKM. Two different sets of primers were used to examine the level of endogenous and total (endogenous plus exogenous) expression for each factor in order to determine transgene-driven contribution. A human BJ-derived iPSC line as well as a human ESC line (H9) was included to compare the levels of expression detected in pluripotent cells to retroviral-transduced cells. Uninfected cells (⊘) were also included as negative control. Results are representative of two independent experiments performed in triplicate.

Ruiz *et al* **Fig S3**

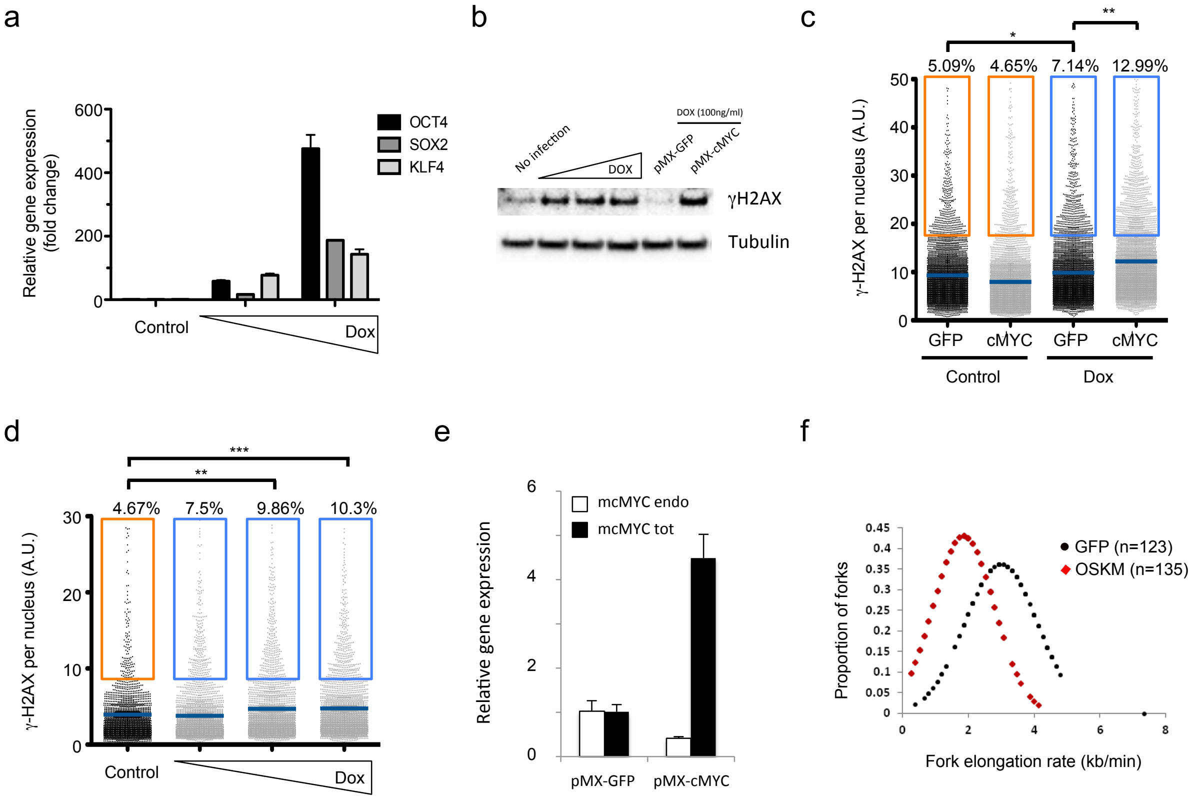

Supplementary Figure 3: Expression of reprogramming factors induces RS. (a) Real-time PCR analysis to quantify relative expression levels of the indicated reprogramming factors. Two different concentrations of doxycycline (100 ng/ml and 500 ng/ml) were added to the growth media for two days at which analysis was performed. Results are representative of two independent experiments performed in triplicate. (b) Western blot analysis of cell extracts obtained from dFib-ind^{OSK} untreated or treated with increasing doses of doxycycline (100, 500 and 1000 ng/ml) for four days to detect γH2AX levels. In addition, cell extracts from dFib-ind^{OSK} infected with retroviruses encoding GFP or c-Myc and treated with doxycycline (100 ng/ml) were also included. See Supplementary Figure 14 for full western blot images. (c) HTM-mediated quantification of γH2AX intensity levels in dFib-ind^{OSK} four days after infection with GFP or cMYC-encoding retroviruses and untreated or treated with doxycycline at a concentration of 100 ng/ml. (d) HTM-mediated quantification of γH2AX intensity levels in dFib-ind^{OSK} four days after treatment with doxycycline at different concentrations (100, 500 and 1000 ng/ml, respectively). For (c and d) center lines indicate mean values whereas squared boxes show the percentage of outliers. Data are representative of two independent experiments performed in triplicate *, p<0.05; **, p<0.01; ***, p<0.001 A.U.= Arbitrary Units. (e) Real-time PCR analysis to quantify the relative expression level of endogenous (endo) and total (tot: endogenous plus exogenous) cMyc in dFib-ind^{OSK} transduced cells. (f) Graphical representation of the fork elongation rate (Kb/min), measured by DNA combing, in BJ fibroblasts four days after infection with retroviruses encoding either GFP or OSKM.

Ruiz *et al* **Fig S4**

a b

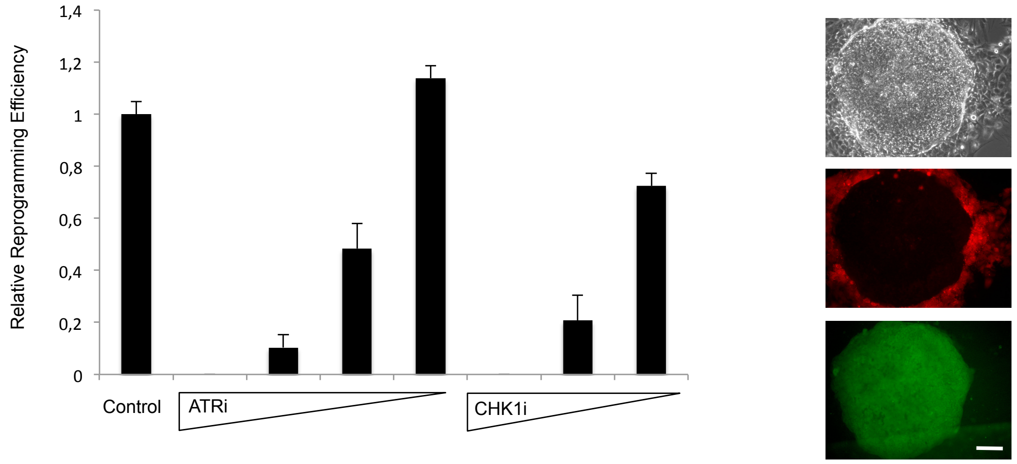

Supplementary Figure 4: Inhibition of ATR and CHK1 interferes with somatic cell reprogramming. (a) Relative reprogramming efficiency determined in dFib-ind^{OSK} cells treated with doxycycline at a concentration of 100 ng/ml. dFib-ind^{OSK} cells were infected with retroviruses encoding a red fluorescent protein (RFP) and cMyc, plated on feeders cells and incubated for three weeks with doxycycline together with ATR or CHK1 inhibitors in a wide range of doses at which the inhibitors were not cytotoxic. Reprogramming efficiency was evaluated by scoring the percentage of GFP positive colonies (resulting from the re-activation of the endogenous OCT4 promoter) and RFP negative colonies (resulting from the silencing of exogenous transgenes). These two features determine the identification of *bona-fide* human iPSC colonies. Data are representative of two independent experiments performed in duplicate. (b) Representative image of an iPSC colony from the experiment shown in (a) showing its morphology (upper panel), silencing of retroviral sequences (middle panel) and reactivation of the OCT4 endogenous promoter (lower panel). Scale bar: 100μM.

Ruiz *et al* **Fig S5**

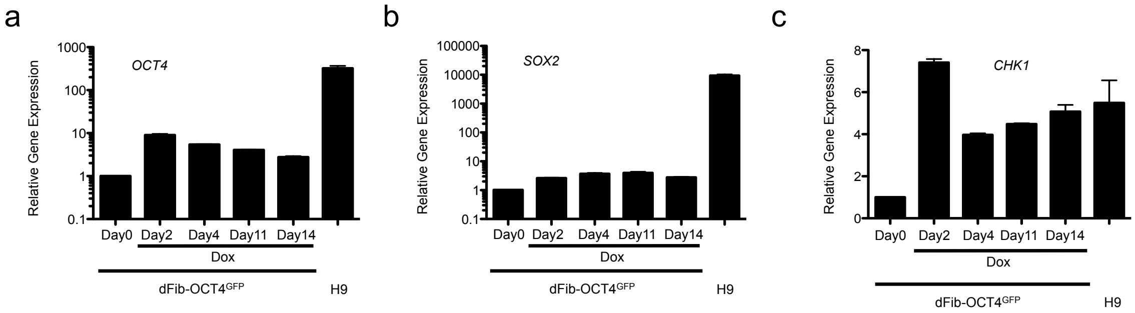

Supplementary Figure 5: Reprogramming factors induce CHK1 transcription. (a-c) Real-time PCR analysis to evaluate gene expression levels of OCT4 (a), SOX2 (b) and CHK1 (c) in dFib-ind^{OSK} cells at different time-points after the addition of doxycycline. Human embryonic stem cells (H9 cells) were used as a reference control. Gene expression values were normalized to GAPDH levels and plotted relative to the expression levels detected at day 0. Data are shown as relative averages ± standard deviation (SD) from two independent experiments performed in triplicate.

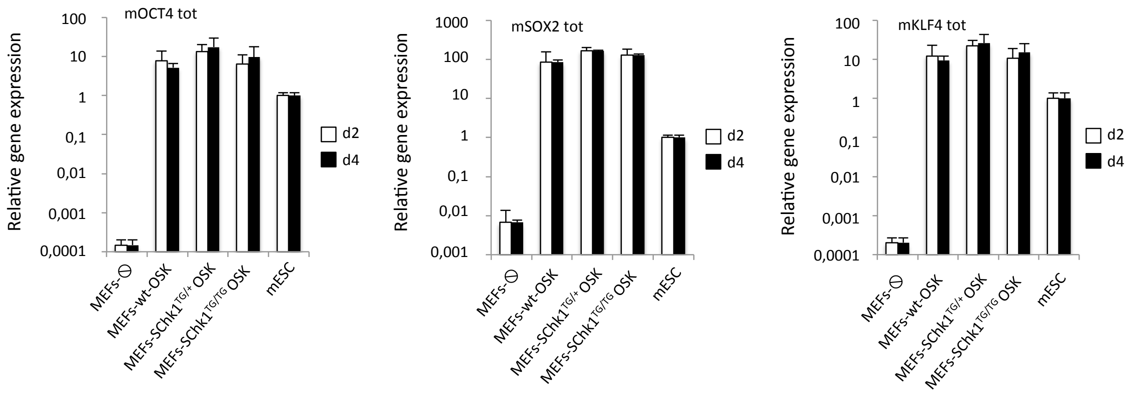

Supplementary Figure 6: Expression of reprogramming factors in wild-type and *Chk1*$^{TG/TG}$ MEF. Real-time PCR analysis to quantify relative expression levels of the indicated reprogramming factors four days after infection with retroviruses. One pair of primers was used to examine the total levels (endogenous plus exogenous). mESC were included to compare the levels of expression detected in pluripotent cells to retroviral-transduced cells. Uninfected cells (⃠) were also included as negative control. Results are representative of two independent experiments performed in triplicate.

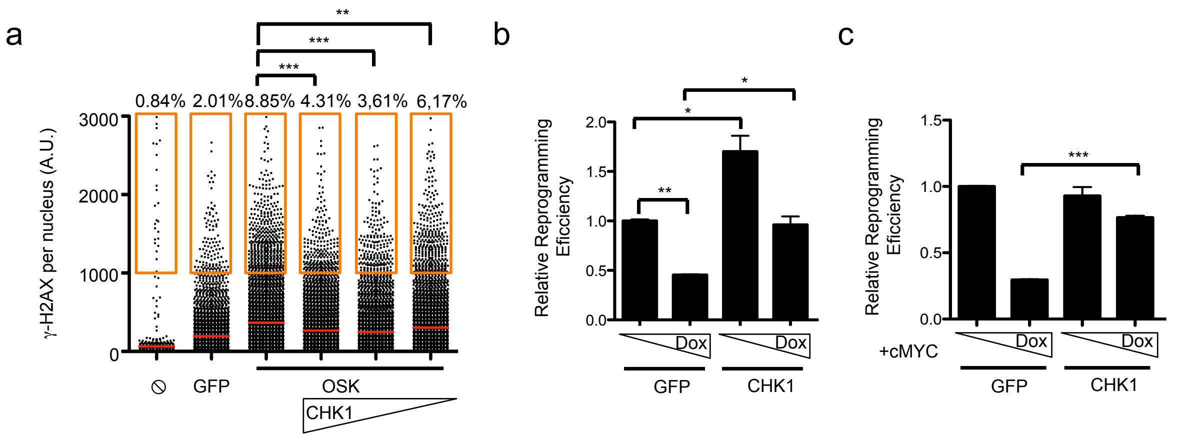

Supplementary Figure 7: CHK1 overexpression increases reprogramming efficiency and reduces reprogramming-induced RS in human fibroblasts. (a) High-throughput microscopy (HTM)-mediated quantification of nuclear γH2AX intensity levels in BJ human fibroblasts four days after infection with retroviruses encoding OSK plus either GFP or different amounts of CHK1. Center lines (in red) indicate mean values whereas squared boxes (in orange) show the percentage of outliers. Data are representative of two independent experiments performed in triplicate ***, $p<0.001$, **, $p<0.01$. Uninfected cells (⦸) were also included as negative control. A.U.= Arbitrary Units. (b) dFib-ind^{OSK} cells were infected with retroviruses encoding either GFP or CHK1 and induced to reprogram. Relative reprogramming efficiencies (evaluated as percentage of Nanog+ colonies) are shown as fold-change normalized to the average efficiency observed in GFP-infected cells. Two different concentrations of doxycycline were used to promote reprogramming (100 ng/ml and 500 ng/ml). Data are representative of two independent experiments performed in triplicate. *, $p<0.05$; **, $p<0.01$. (c) dFib-ind^{OSK} cells were infected with retroviruses encoding cMYC and either GFP or CHK1. Relative reprogramming efficiencies (evaluated as percentage of Nanog+ colonies) are represented as fold change normalized to the average efficiency observed in GFP-infected cells. Two different concentrations of doxycycline were used to promote reprogramming (100 ng/ml and 500 ng/ml). Data are representative of two independent experiments performed in triplicate. ***, $p<0.001$.

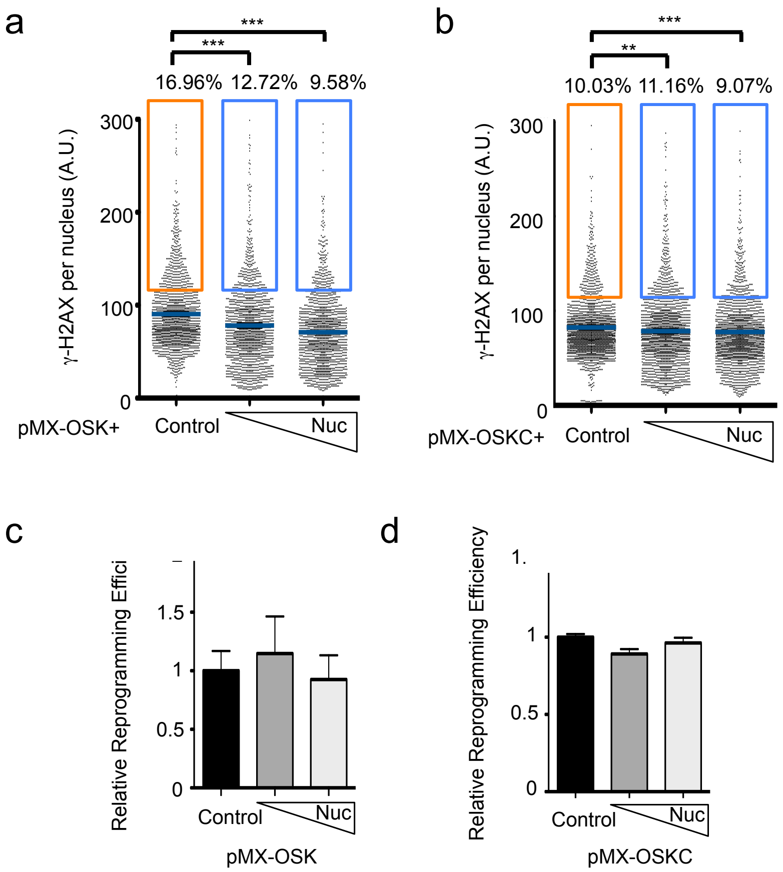

Supplementary Figure 8: Effect of nucleoside supplementation on reprogramming-induced RS and reprogramming efficiency. (a, b) HTM-mediated quantification of γH2AX intensity levels in BJ human fibroblasts four days after infection with OSK (a) or OSKM (b) with or without daily addition of nucleosides. Center lines indicate mean values whereas squared boxes show the percentage of outliers. Data are representative of three independent experiments performed in triplicate **, $p<0.01$; ***, $p<0.001$. A.U.= Arbitrary Units. (c) BJ fibroblasts were infected with retroviruses encoding OSK (left panel) and OSKM (right panel) and either untreated or treated with different amounts of nucleosides (0.5X and 2X). Relative reprogramming efficiencies (evaluated as percentage of Nanog+ colonies) are shown as fold change normalized to the average efficiency observed untreated cells. Data are representative of two independent experiments performed in triplicate.

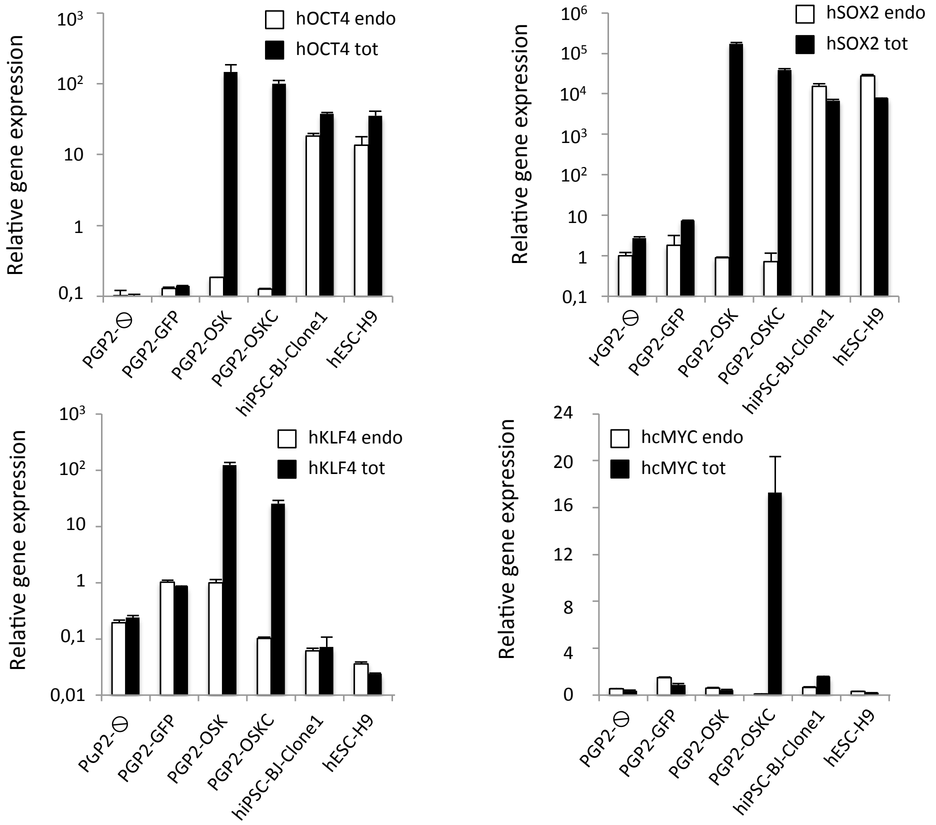

Supplementary Figure 9: Expression of reprogramming factors in PGP2 human fibroblasts. Real-time PCR analysis to quantify relative expression levels of the indicated reprogramming factors four days after infection with retroviruses encoding GFP, OSK or OSKM. Two different sets of primers were used to examine the level of endogenous and total (endogenous plus exogenous) expression for each factor in order to determine transgene-driven contribution. A human BJ-derived iPSC line as well as a human ESC line was included to compare the levels of expression detected in pluripotent cells to retroviral-transduced cells. Uninfected cells (⊘) were also included as negative control. Results are representative of two independent experiments performed in triplicate.

Ruiz *et al* **Fig S10**

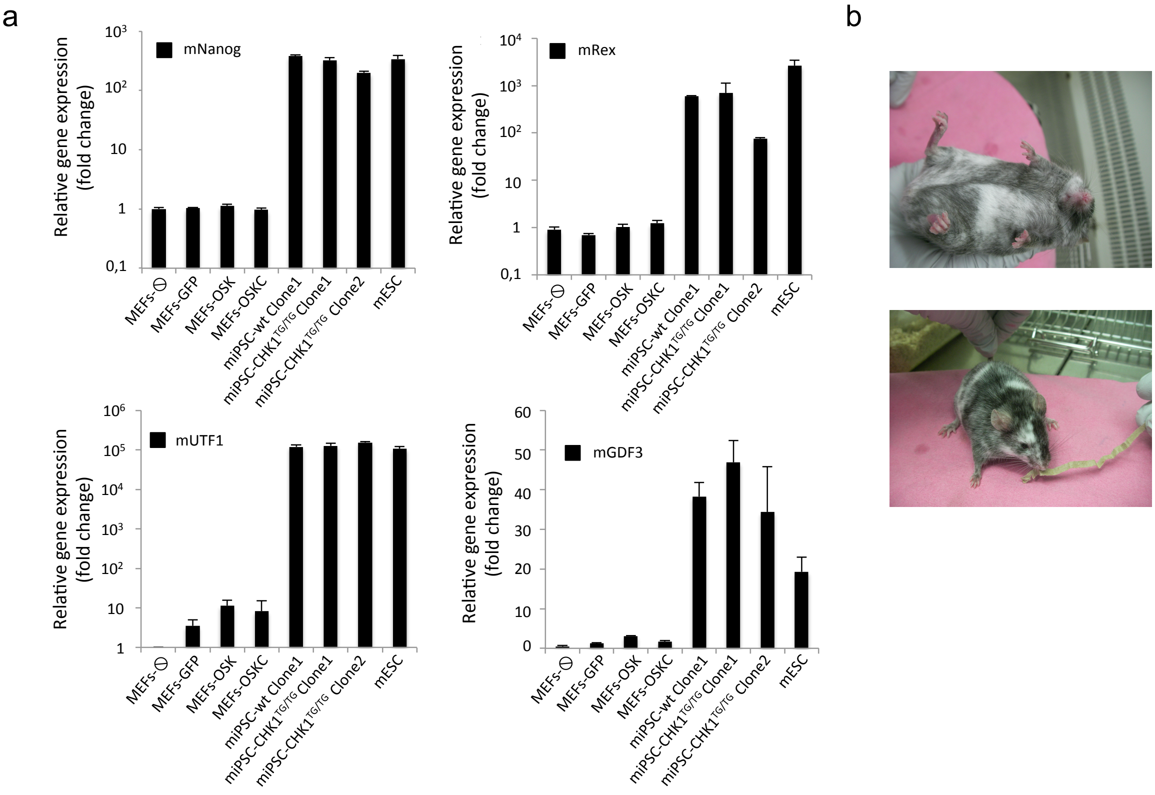

Supplementary Figure 10: Pluripotency of wt and *Chk1*^{TG} iPSC. (a) Real-time PCR analysis to quantify relative expression levels of the indicated pluripotent factors (Nanog, Rex, UTF1 and GDF3) in different mouse iPSC lines. mESC as well as MEF infected with retrovirus expressing the reprogramming factors for a total of four days were included to compare the levels of expression detected in pluripotent cells to retroviral-transduced cells. Uninfected cells (⊘) were also included as negative control. Results are representative of two independent experiments performed in triplicate. (b) Representative images of chimeric mice generated with *Chk1*^{TG} iPSC. Two different images are shown to demonstrate the level of chimerism in the generated mice.

Ruiz *et al* **Fig S11**

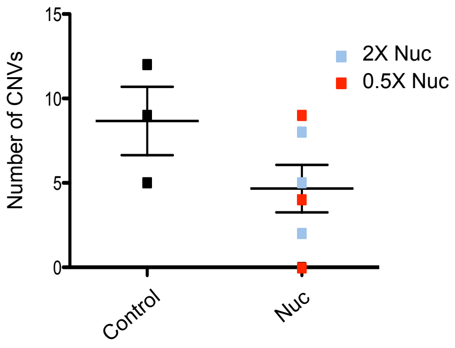

Supplementary Figure 11: Nucleoside supplementation decreases genomic instability on iPSC. SurePrint G3 Human High Resolution Microarrays were used to analyze the number of CNV in hiPSC clones untreated or treated with different amounts (0.5X and 2X) of nucleosides during the whole reprogramming process. Data presented here are from an independent experiment to those represented on Figure 4. A one-tailed unpaired t-test was used to compare CNV datasets. P = 0.07.

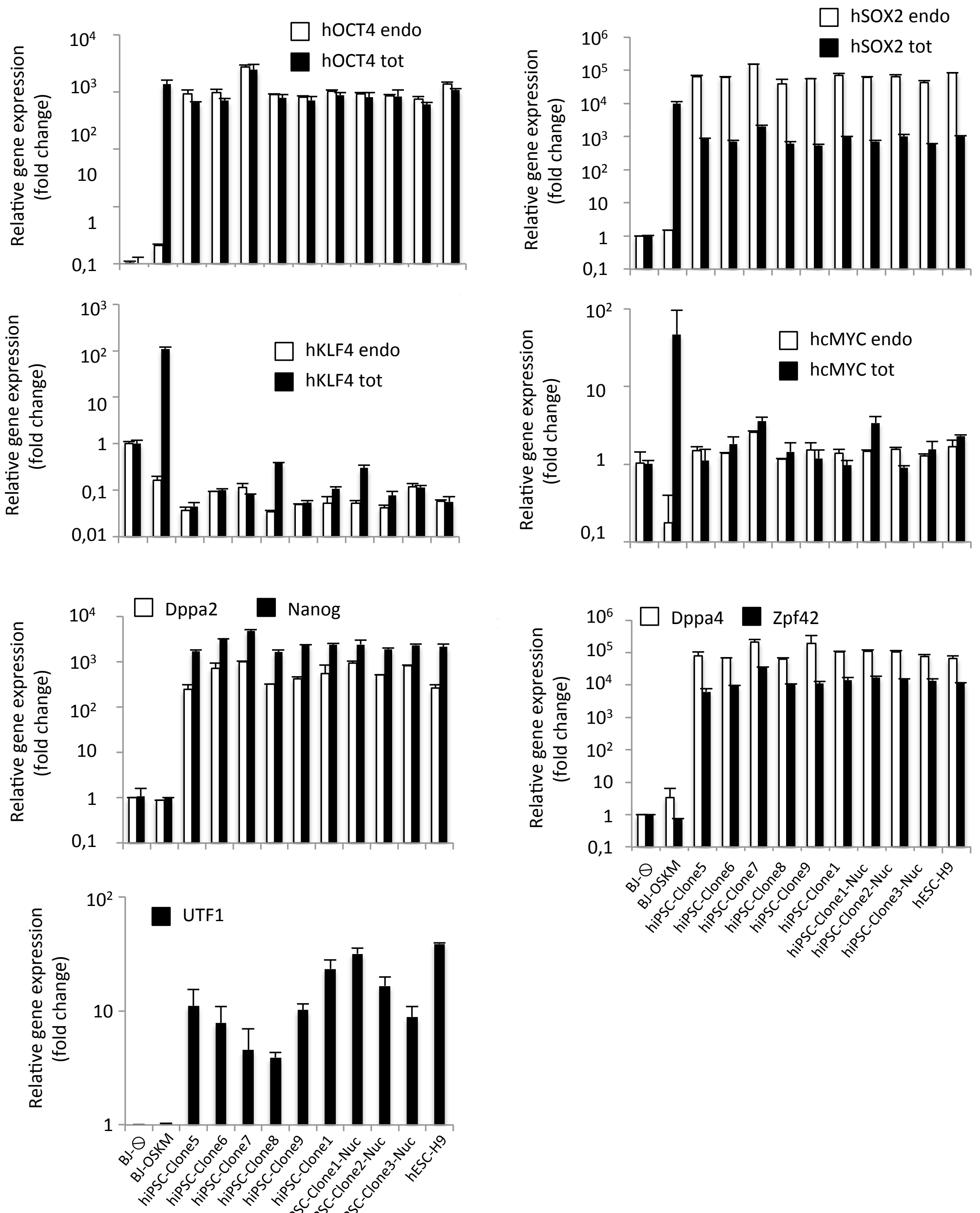

Supplementary Figure 12: Expression of pluripotent factors in hiPSC lines. Real-time PCR analysis to quantify relative expression levels of the indicated pluripotent factors in different human iPSC lines. For detecting OCT4, SOX2, KLF4 and cMYC, two different sets of primers were used to examine the level of endogenous (endo) and total (tot: endogenous plus exogenous) expression for each factor in order to determine transgene-driven contribution. hESC (H9) as well as BJ fibroblasts infected with retrovirus expressing the reprogramming factors for a total of four days were included to compare the levels of expression detected in pluripotent cells to retroviral-transduced cells. Uninfected cells (⃠) were also included as negative control. Expression of the pluripotent factors Dppa2, Dppa4, Nanog, Zpf42 and UTF1 was used to demonstrate the pluripotency of the generated hiPSC lines. Results are representative of two independent experiments performed in triplicate.

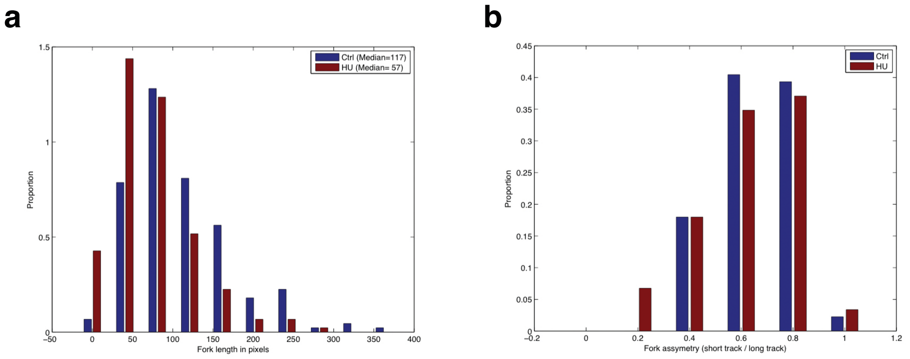

Supplementary Figure 13: Reduced fork length in the absence of fork asymmetry in response to HU. DNA combing was performed in U2OS exposed or not to HU (1 mM, 2 hrs). More than 100 fibers were analyzed per condition. The impact of HU on fork length (a) and fork asymmetry (b) are shown. Whereas HU treatment approximately halved fork lengths (Ctrl: 132.8 ± 63.8, HU: 65.7 ± 41.7; p=1.3e-29), it had no significant impact on fork asymmetry (Ctrl: 0.76 ± 0.15, HU: 0.72 ± 0.18; p=0.37).

Ruiz *et al* **Fig S14**

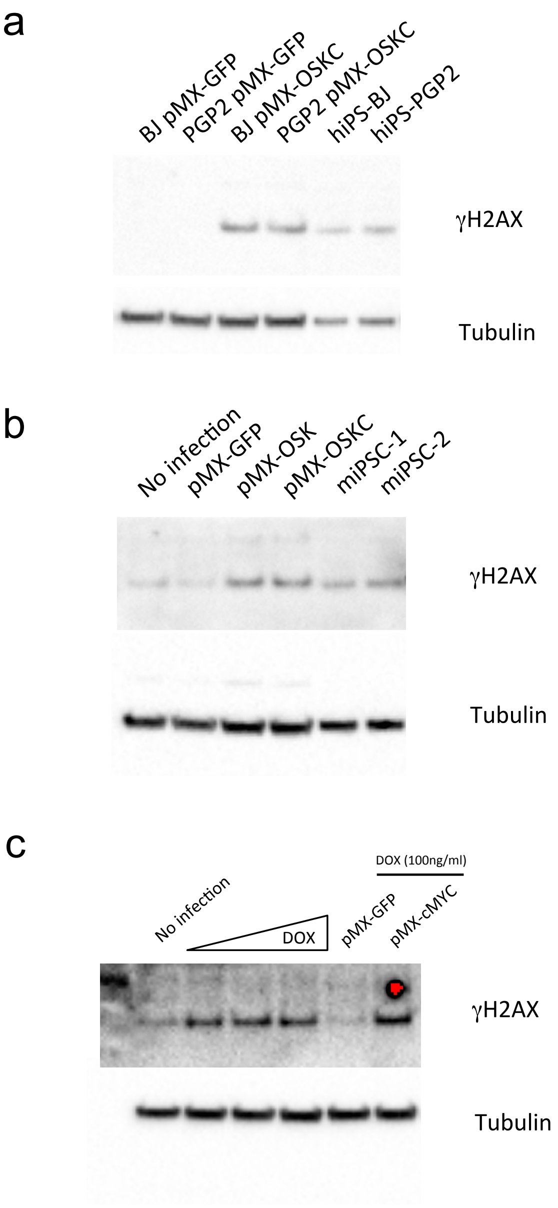

Supplementary Figure 14: (a-c) Full western blot images corresponding to Figures 1d (a), 1b (b) and Supplementary Figure 3b (c).

Supplementary Table 1: List of CNVs detected in human iPSC from Fig. 4.

Chr	Start-Stop(bp)	Cytoband	Amp/Gain/Loss/Del	Annotations	Description	Ref
hiPSC-1						
chr1	8889520-8978531	p36.23	0.261315	ENO1	Most common terminal deletion syndrome in humans; Aphidicolin-induced CNV	1,2
chr1	56027023-56106359	p32.3-p32.2	-0.297285		Found in microdeletions	3
chr8	43169025-43315591	p11.1	-0.406213		Found in chromosomal translocations	4
chr11	64067876-64113149	q13.1	0.567597	C11orf20, ESRRA, TRMT112...	Found in microdeletions	5
hiPSC-2						
chr6	16403105-16621631	p22.3	-0.991262	ATXN1	CNV associated with autism spectrum disorders; HU-induced CNV; known fragile site	2,6,7
chr6	99848079-99926180	q16.2	0.267289	SFRS18, USP45	Found in microdeletions	8
hiPSC-3						
chr3	187905111-188427125	q28	-1.032032	LPP		
chr9	130143585-130175156	q33.3	0.620317	GARNL3, SLC2A8	Recurrent CNV in autism; HU-induced CNV	9,2
chr14	66914314-67048022	q23.3	-0.963191	NCRNA00238, GPHN	Microdeletion in autism; known fragile site	10,7
hiPSC-4						
chr1	72636263-72879544	p31.1	-0.982252	NEGR1	FRA1C	11
chr6	149637723-149673588	q25.1	0.362086	TAB2	Found in microdeletions	12
chr11	65181037-65274819	q13.1	0.268871	NEAT1, MIR612, MALAT1	Chromosomal rearrangements frequent in cancer	13
chrX	7125588-7237083	p22.31	-0.560013	STS	Chromosomal rearrangements in behavioral disorders; known fragile site	14,7
chrX	64879847-64897793	q12	0.489425	MSN	CNV found in autism	15
hiPSC-1 Nuc						
chr16	78999781-79065417	q23.1	-0.889569	WWOX	FRA16D	16,7

hiPSC-5						
chr16	78574950-78757037	q23.1	-0.548514	WWOX	FRA16D	16,7
chr20	60566112-60573500	q13.33	0.680129	TAF4		
hiPSC-6						
chr1	72523846-72539623	p31.1	-0.910923	NEGR1	FRA1C	11
hiPSC-7						
chr1	245409995-245985973	q44	0.427073	KIF26B, SMYD3	Found in microdeletions; HU-induced CNV	17,2
chr5	14399169-14525275	p15.2	0.506479	TRIO	CNV found in Cat cry syndrome; HU-induced CNV	18,2
chr6	5190407-5225627	p25.1	-0.835361	LYRM4	Found in microdeletions; known fragile site	19,7
chr15	86407812-86427500	q25.3	-0.854835		HU-induced CNV	2
hiPSC-8						
chr1	85580363-85952762	p22.3	0.623118	WDR63, SYDE2, C1orf52...	Found in microdeletions; HU-induced CNV; known fragile site	20,7
chr3	29459879-29811951	p24.1	-1.048445	RBMS3	Breakpoint in Marfan syndrome	21
chr10	104688674-105365526	q24.32 - q24.33	-0.843053	CNNM2, NT5C2, LOC729020...		
chr12	11112235-11336621	p13.2	-0.936613	PRH1-PRR4, TAS2R50, TAS2R20...	Frequently rearranged in cancer	22
chrY	18384877-18787276	q11.221	0.835503			
hiPSC-9						
chr3	78724066-79011383	p12.3	-0.416846	ROBO1	Known fragile site; Aphidicolin-induced CNV	10,2
chr5	14399169-14530635	p15.2	0.497294	TRIO	CNV found in Cat cry syndrome; HU-induced CNV	18,2
chr6	87666768-87680005	q14.3	0.620713	HTR1E	Found in microdeletions; HU-induced CNV	23
chr15	86407812-86427500	q25.3	-0.898797		HU-induced CNV	2
chr16	78620813-78641435	q23.1	-0.575855	WWOX	FRA16D	16,7
hiPSC-2-Nuc						
chr12	891288-1350728	p13.33	-0.838066	WNK1, RAD52, ERC1	Found in microdeletions; HU-induced CNV	24,2

hiPSC-3-Nuc						
chr6	84476255-84689214	q14.2	-0.858336	RIPPLY2, CYB5R4	Found in microdeletions	25
chr8	97876845-97996457	q22.1	-0.275527	PGCP	Aphidicolin-induced CNV	26
chrY	28460973-28779402	q11.23	0.494045		Found in microdeletions	27
hiPSC-4-Nuc						
chr1	198199363-198278461	q31.3	0.529572	NEK7	HU-induced CNV; integration site for HPV16	2, 28,29
chr16	82737224-82892105	q23.3	0.479920	CDH13	Known CFS; Deletion in ovarian cancer; HU- and Aphidicolin-induced CNV	11,30,2,7
chrX	62864366-63066833	q11.1 - q11.2	0.949275	ARHGEF9, MIR1468	73% of miRNAs locate to fragile sites	31
hiPSC-5-Nuc						
chr13	61436625-61892934	q21.2	0.496511	MIR3169	73% of miRNAs locate to fragile sites; HU-induced CNV; known fragile site	31,2,7
chr20	26148870-26200119	p11.1	0.412487	MIR663	73% of miRNAs locate to fragile sites	31

Supplementary Table 2: List of CNVs detected in human iPSC from Supplementary Fig.11.

Chr	Start-Stop(bp)	Cytoband	Type	Description	Reference
hiPSC-1					
chr10	134599944-134604551	q26.3	LOSS	HU-induced CNV; observed CFS	32,33
chr10	134893462-124898682	q26.3	LOSS	HU-induced CNV	32,33
chr16	2166410-2169029	p13.3	LOSS	CNV found in Rubinstein-Taybi syndrome	34
chr22	20130600-20136727	q11.21	LOSS	Involved in 22q11.2 duplication syndrome	35
chr4	1550584-1554704	p16.3	LOSS	CNV involved in Wolf-Hirchhorn syndrome; known fragile site	36,33
hiPSC-2					
chr18	29072578-30655180	q12.1	GAIN	CNV involved in autism	37
chr10	57251916-57256006	q21.1	LOSS	HU-induced CNV	32
chr10	134889444-134898682	q26.3	LOSS	HU-induced CNV; known fragile site	32,33
chr12	27655270-17660764	p11.23	LOSS		
chr12	129124426-129132044	q24.32	LOSS		
chr16	2166346-2169029	p13.3	LOSS		
chr4	1550584-1554704	p16.3	LOSS	CNV involved in Wolf-Hirchhorn syndrome; known fragile site	36,33
chr6	63112232-63117244	q11.1	LOSS		
chr6	163064654-163530458	q26	LOSS	Found in microdeletions; FRA6E	39,33
chr7	16205662-16210866	p21.2	LOSS	Involved in duplications	38
chr7	57538824-57544688	p11.2	LOSS	Involved in deletions	40
chr7	69567984-69575722	q11.22	LOSS	HU-induced CNV	32
hiPSC-3					
chr1	246060296-246176068	q44	LOSS	APH and HU-induced CNV; known fragile site	32,33
chr10	68627002-68634102	q21.3	LOSS	Involved in deletions	41
chr14	104787582-104792234	q32.33	LOSS	HU-induced CNV	32
chr14	106200027-106204834	q32.33	LOSS	HU-induced CNV	32

chr16	78065914-78124166	q23.1	LOSS	APH and HU-induced CNV; known fragile site	32,33
chr4	1420890-1496182	p16.3	LOSS	CNV involved in Wolf-Hirchhorn syndrome; known fragile site	36,33
chr6	103748758-103753910	q16.3	LOSS	APH and HU-induced CNV	32
chr12	57301660-57785494	q13.3	GAIN	Amplification in cancer	42
chr3	172642768-188602818	q26.31	GAIN	APH and HU-induced CNV	32
hiPSC-4 2XNuc					
chr10	38789636-38885064	p11.1	LOSS	HU-induced CNV	32
chr15	91023680-91075556	q23.31	LOSS		
chr16	34220999-466229978	p11.2	LOSS	Involved in deletions	43
chr2	90374370-90384758	p11.2	LOSS	Involved in deletions	44
chr7	78038890-78351907	q21.11	LOSS	APH and HU-induced CNV	32
hiPSC-5 2XNuc					
chr2	61591122-62158932	p15	GAIN	Involved in deletions	45
chr6	114188024-128961866	q21	GAIN	Involved in deletions; known fragile site	46,33
hiPSC-6 2XNuc					
chr3	62210610-62761898	p14.2	GAIN	FRA3B; HU-induced CNV; integration site for HPV16	32,33, 47
chr10	39127651-42062507	p11.1	LOSS	HU-induced CNV	32
chr11	4040482-4272842	p15.4	LOSS	Involved in microduplications	48
chr12	75260598-75265804	q21.1	LOSS	Identified as CNV	49
chr13	69664684-90697850	q21.33	LOSS	Involved in deletions	50
chr19	31984460-31993178	q12	LOSS		
chr19	42419329-42426954	q13.2	LOSS	Involved in deletions	51
chr7	1306028-1311270	p22.3	LOSS	Involved in deletions; FRA3B	52,33
hiPSC-7 0.5XNuc					
chr13	114773092-114776596	q34	GAIN		
chr22	19710530-19714616	q11.21	GAIN	Involved in 22q11.2 duplication syndrome	35

chr3	60501728-61070084	p14.2	GAIN	FRA3B; HU-induced CNV; integration site for HPV16	33,47
chr13	93887108-94111768	q31.3	LOSS	HU-induced CNV	32
hiPSC-9 0.5XNuc					
chr1	1295361-1303058	p36.33	LOSS		
chr10	39127651-39139010	p11.1	LOSS	HU-induced CNV	32
chr11	1883542-1892398	p15.5	LOSS		
chr19	21055950-21073538	p12	LOSS		
chr2	90371587-92272016	p11.2	LOSS	Involved in deletions	44
chr2	132512464-132565372	q21.2	LOSS		
chr22	16050404-17013601	q11.1	LOSS		
chr22	50606493-50618692	q13.33	LOSS		
chr4	49163568-49168398	p11	LOSS		

Supplementary Table 3: Point somatic variants identified via exome sequencing on iPSC.

Sample Name	Num Variants	Num known (dbSNP)	Non-parental Variants	Potential Somatic Variants	Affected genes
FiPS-1	11,990	11,188	141	10	FBXW10, KBTBD4, QRICH2, MUC4, TPTE, CYLC2, NDUFS3, SLC6A15
FiPS-2	12,077	11,218	135	13	LST3, HADHB, IFI27, BCLAF1, SLCO1B3, MUC20, SLCO1B7, GPR115, RNF216, GPR111
FiPS-3	12,027	11,199	146	10	PABPC1, AQP7, VWF, SPAG11A, FCGBP
FiPS-4	12,063	11,220	149	14	FAM131C, C21orf128, MST1, LILRA6, UMODL1, SIGLEC11, APEH, MUC16, PLIN4, RNF123, VWF
FiPS-5	12,019	11,204	141	7	MUC4
nuc-FiPS-1	12,085	11,275	141	3	FCGBP
nuc-FiPS-2	12,002	11,223	153	8	DPAGT1, C2CD2L, ELAVL3, CES1, PRKCSH, FBXO25
nuc-FiPS-3	12,054	11,237	176	6	CYP2A7, MUC6
nuc-FiPS-4	12,025	11,227	193	11	CASP1, FAM120B, PABPC1, NUP160, MAP2K3, AXDND1, RGPD4, LILRA6, MUC5B
nuc-FiPS-5	12,022	11,225	149	7	SIRPA, SRA1, EIF4EBP3, ANKHD1, MUC16, SLC6A15
HFF	11,964	11,168	NA	NA	NA

Note: The elevated frequency of point mutations observed in mucin genes is a frequent observation in exome sequencing datasets (see, for instance, ref 53). One of the current interpretations is that because mucins are recent gene copy events they give rise to frequent paralogous alignments, which in turn might lead to false-positive variant calling (for a discussion on this, please see http://massgenomics.org/2013/06/ngs-false-positives.html). Since we cannot formally discard that these are bona-fide mutations, they have been included with the rest of the identified variants. Nevertheless, not considering the mucins would not change the message that the nucleoside supplementation described here is not mutagenic.

Supplementary Table 4: List of primers used in this work.

Primer name	Sequence 5’-3’
mOCT4endo-F	TCTTTCCACCAGGCCCCCGGCTC
mOCT4endo-R	TGCGGGCGGACATGGGGAGATCC
mSOX2endo-F	TAGAGCTAGACTCCGGGCGATGA
mSOX2endo-R	TTGCCTTAAACAAGACCACGAAA
mKLF4endo-F	GCGAACTCACACAGGCGAGAAACC
mKLF4endo-R	TCGCTTCCTCTTCCTCCGACACA
mcMYCendo-F	TGACCTAACTCGAGGAGGAGCTGGAATC
mcMYCendo-R	AAGTTTGAGGCAGTTAAAATTATGGCTGAAGC
mOCT4total-F	CTGAGGGCCAGGCAGGAGCACGAG
mOCT4total -R	CTGTAGGGAGGGCTTCGGGCACTT
mSOX2total -F	GGTTACCTCTTCCTCCCACTCCAG
mSOX2total -R	TCACATGTGCGACAGGGGCAG
mKLF4total -F	CACCATGGACCCGGGCGTGGCTGCCAGAAA
mKLF4total -R	TTAGGCTGTTCTTTTCCGGGGCCACGA
mcMYCtotal -F	CAGAGGAGGAACGAGCTGAAGCGC
mcMYCtotal -R	TTATGCACCAGAGTTTCGAAGCTGTTCG
hOCT4endo-F	GGGTTTTTGGGATTAAGTTCTTCA
hOCT4endo-R	GCCCCCACCCTTTGTGTT
hSOX2endo-F	CAAAAATGGCCATGCAGGTT
hSOX2endo-R	AGTTGGGATCGAACAAAAGCTATT
hKLF4endo-F	AGCCTAATTGATGGTGCTTGGT
hKLF4endo-R	TTGAAAACTTTGGCTTCCTTGTT
hcMYCendo-F	CGGGCGGGCACTTTG
hcMYCendo-R	GGAGAGTCGCGTCCTTGCT
hOCT4total-F	GGAGGAAGCTGACAACAATGAAA
hOCT4total -R	GGCCTGCACGAGGGTTT
hSOX2total -F	TGCGAGCGCTGCACAT
hSOX2total -R	TCATGAGCGTCTTGGTTTTCC
hKLF4total -F	CGAACCCACACAGGTGAGAA
hKLF4total -R	GAGCGGGCGGCGAATTTCCAT
hcMYCtotal -F	AGGGTCAAGTTGGACAGTGTCA
hcMYCtotal -R	TGGTCGATTTTCGGTTGTTG
LentiOCT4-F	CCCCTGTCTCTGTCACCACT
LentiOCT4-R	CCACATAGCGTAAAAGGAGCA
LentiSOX2-F	ACACTGCCCCTCTCACACAT
LentiSOX2-R	CATAGCGTAAAAGGAGCAACA
LentiKLF4-F	GACCACCTCGCCTTACACAT
LentiKLF4-R	CATAGCGTAAAAGGAGCAACA
mNanog-F	CAGGTGTTTGAGGGTAGCTC
mNanog-R	CGGTTCATCATGGTACAGTC
mRex-F	ACGAGTGGCAGTTTCTTCTTGGGA
mRex-R	TATGACTCACTTCCAGGGGGCACT

mUTF1-F	GGATGTCCCGGTGACTACGTCTG
mUTF1-R	GGCGGATCTGGTTATCGAAGGGT
mGDF3-F	GTTCCAACCTGTGCCTCGCGTCTT
mGDF3-R	AGCGAGGCATGGAGAGAGCGGAGCAG
hDppa2-F	CTGGTGCCAGTTAAAGATGACG
hDppa2-R	TGTGGAGCTGTAAATTGCTCATT
hDppa4-F	TCCTGGGCGAGAATTTCAGC
hDppa4-R	GCAGGTGAACCCAACCATCT
hNanog-F	ACAACTGGCCGAAGAATAGCA
hNanog-R	GGTTCCCAGTCGGGTTCAC
hZpf42-F	ACCGGGCAAAGACAAGACAC
hZpf42-R	GCTGACAGGTTCTATTTCCGC
hUTF1-F	CGCCGCTACAAGTTCCTTAAA
hUTF1-R	GGATCTGCTCGTCGAAGGG
GAPDH-F	GGACTCATGACCACAGTCCATGCC
GAPDH-R	TCAGGGATGACCTTGCCCACAG

SUPPLEMENTARY REFERENCES:

1) Heilstedt, H. A., et al. Population data suggest that deletions of 1p36 are a relatively common chromosome abnormality. *Clin. Genet.* **64**: 310-316, (2003).

2) Arlt, M.F. et al. Hydroxyurea induces de novo copy number variants in human cells. Proc. Natl. Acad. Sci. USA. **108:** 17360-17365 (2011).

3) Koehler, U., et al. A novel 1p31.3p32.2 deletion involving the NFIA gene detected by array CGH in a patient with macrocephaly and hypoplasia of the corpus callosum. *Eur J Pediatr.* **169**: 463-8 (2010).

4) Jackson, C.C., Medeiros, L.J. and Miranda, R.N. 8p11 myeloproliferative syndrome: a review. *Hum Pathol.* **41**: 461-76 (2010).

5) Ferreira de Almeida, T. and Bertola, D.R. Microdeletion 11q13.1.q13.2 in a patient presenting with developmental delay, facial dysmorphism, and esophageal atresia: Possible role of the GSTP1 gene in esophagus malformation. *Birth Defects Res A Clin Mol Teratol.* **97**: 463-6 (2013).

6) Celestino-Soper, P.B. et al. Deletions in chromosome 6p22.3-p24.3, including ATXN1, are associated with developmental delay and autism spectrum disorders*. Mol Cytogenet.* **5**:17 (2012).

7) Durkin, S.G. and Glover, T.W. Chromosome fragile sites. *Annu Rev Genet.* **41**: 169-92 (2007).

8) Klein, O.D. et al. Interstitial deletions of chromosome 6q: genotype-phenotype correlation utilizing array CGH. *Clin Genet.* **71**: 260-6 (2007).

9) Sanders , S.J. et al. Multiple recurrent de novo CNV, including duplications of the 7q11.23 William syndrome region, are strongly associated with autism. *Neuron.* **70**: 863-85 (2011).

10) Griswold, A.J. et al. A de novo 1.5 Mb microdeletion on chromosome 14q23.2-23.3 in a patient with autism and spherocytosis. *Autism Res.* **4**: 221–227 (2011).

11) Thomas, E. et al. Large transcription units unify copy number variants and common fragile sites arising under replication stress. *Genome Res.* **25**: 189–200 (2015).

12) Sukumar, S., et al. Subtle overlapping deletions in the terminal region of chromosome 6q24.2-q26: three cases studied using FISH. *Am J Med Genet.* **87**: 17-22 (1999).

13) Lammie, G.A. and Peters, G. Chromosome 11q13 abnormalities in human cancer. *Cancer Cells* **3**: 413-20 (1991).

14) Liu, P. et al. Copy number gain at Xp22.31 includes complex duplication rearrangements and recurrent triplications. *Hum Mol Genet.* **20**: 1975-88 (2011).

15) Kaya, N. et al. A novel X-linked disorder with developmental delay and autistic features. *Ann Neurol*. **71**: 498-508 (2012).

16) Finnis, M. et al. Common chromosomal fragile site FRA16D mutation in cancer cells. *Hum Mol Genet.* **14**: 1341-9 (2005).

17) Boland, E. et al. Mapping of deletion and translocation breakpoints in 1q44 implicates the serine/threonine kinase AKT3 in postnatal microcephaly and agenesis of the corpus callosum. *Am J Hum Genet.* **81**: 292-303 (2007).

18) Jones KL Deletion 5p Syndrome. In K.L. Jones (6th Edition), Smith's Recognizable Patterns of Human Malformation. (pp. 40-41). Philadelphia: Elsevier. (2006).

19) Suzuki, K. et al. Case of chromosome 6p25 terminal deletion associated with Axenfeld-Rieger syndrome and persistent hyperplastic primary vitreous. *Am J Med Genet A.* **140**: 503-8 (2006).

20) Tabata, H. et al. Short arm deletion of chromosome 1: del(1)(p13.3 p22.3) in a female infant with an extreme tetralogy of Fallot. *Clin Genet*. **39**: 132-5 (1991).

21) Mizuguchi, T. et al. Heterozygous TGFBR2 mutations in Marfan syndrome. *Nat Genet*. **36**: 855-60 (2004).

22) Sato, Y. et al. Chromosomal instability in chromosome band 12p13: multiple breaks leading to complex rearrangements including cytogenetically undetectable sub-clone. *Leukemia*. **15**: 1193-202 (2001).

23) Becker, K. et al. De novo microdeletions of chromosome 6q14.1-q14.3 and 6q12.1-q14.1 in two patients with intellectual disability - further delineation of the 6q14 microdeletion syndrome and review of the literature. *Eur J Med Genet.* **55**: 490-7 (2012).

24) Thevenon, J. et al. 12p13.33 microdeletion including ELKS/ERC1, a new locus associated with childhood apraxia of speech. *Eur J Hum Genet.* **21**: 82-8 (2013).

25) Wentzel, C. et al. Interstitial Deletions at 6q14.1-q15 Associated with Obesity, Developmental Delay and a Distinct Clinical Phenotype. *Mol Syndromol.* **1**:75-81 (2010).

26) Austin, M.J. et al. Aphidicolin-inducible common fragile-site expression: results from a population survey of twins. *Am J Hum Genet*. **50**: 76-83 (1992).

27) Weise, A. et al. Microdeletion and microduplication syndromes. *J Histochem Cytochem*. **60**: 346-58 (2012).

28) Thorland, E.C. et al. Common fragile sites are preferential targets for HPV16 integrations in cervical tumors. *Oncogene* **22**: 1225–37 (2003).

29) Wilke, C.M. et al. FRA3B extends over a broad region and contains a spontaneous HPV16 integration site: direct evidence for the coincidence of viral integration sites and fragile sites. *Human Molecular Genetics* **5**: 187–95 (1996).

30) Kawakami, M. et al. Involvement of H-cadherin (CDH13) on 16q in the region of frequent deletion in ovarian cancer. *Int J Oncol*. **15**: 715-20 (1999).

31) Calin, G.A. et al. Human microRNA genes are frequently located at fragile sites and genomic regions involved in cancers. *Proc Natl Acad Sci U S A.* **101**: 2999-3004 (2004).

32) Arlt, M.F. et al. Hydroxyurea induces de novo copy number variants in human cells. Proc. Natl. Acad. Sci. USA. **108:** 17360-17365 (2011).

33) Durkin, S.G. and Glover, T.W. Chromosome fragile sites. *Annu Rev Genet*. **41**: 169-92 (2007).

34) Hennekam, R. C., et al. Deletion at chromosome 16p13.3 as a cause of Rubinstein-Taybi syndrome: clinical aspects. *Am J Hum Genet.* **52**: 255–262 (1993).

35) Wentzel, C. et al. Clinical variability of the 22q11.2 duplication syndrome. *Eur J Med Genet*. **51**: 501-10 (2008).

36) Van Buggenhout, G. et al. Mild Wolf-Hirschhorn syndrome: micro-array CGH analysis of atypical 4p16.3 deletions enables refinement of the genotype-phenotype map. *J Med Genet* **41**:691-698 (2004).

37) Wang, P. et al. Genotype-phenotype analysis of 18q12.1-q12.2 copy number variation in autism. *Eur J Med Genet*. **56**: 420-5 (2013).

38) Cai, T. et al. Duplication of 7p21.2-->pter due to maternal 7p;21q translocation: implications for critical segment assignment in the 7p duplication syndrome. *Am J Med Genet*. 86: 305-11 (1999).

39) Sukumar, S., et al. Subtle overlapping deletions in the terminal region of chromosome 6q24.2-q26: three cases studied using FISH. *Am J Med Genet.* **87**: 17-22 (1999).

40) Varvagiannis, K. et al. De novo 393 kb microdeletion of 7p11.2 characterized by aCGH in a boy with psychomotor retardation and dysmorphic features. *Meta Gene*. **2**: 274-82 (2014).

41) Kim, J.H. et al. Deletion variants of RABGAP1L, 10q21.3, and C4 are associated with the risk of systemic lupus erythematosus in Korean women. *Arthritis Rheum*. **65**: 1055-63 (2013).

42) Park, S. et al. Aberrant CDK4 amplification in refractory rhabdomyosarcoma as identified by genomic profiling. *Sci Rep*. **4**:3623 (2014).

43) Hempel, M. et al. Microdeletion syndrome 16p11.2-p12.2: clinical and molecular characterization. *Am J Med Genet A*. **149**: 2106-12 (2009).

44) Tzschach, A. et al. Interstitial deletion 2p11.2-p12: report of a patient with mental retardation and review of the literature. *Am J Med Genet A.* **149**: 242-5 (2009).

45) Piccione, M. et al. Interstitial deletion of chromosome 2p15-16.1: report of two patients and critical review of current genotype-phenotype correlation. *Eur J Med Genet.* **55**: 238-44 (2012).

46) Hudson, C. et al. Confirmation of 6q21-6q22.1 deletion in acro-cardio-facial syndrome and further delineation of this contiguous gene deletion syndrome. *Am J Med Genet A.* 164: 2109-13 (2014).

47) Wilke, C.M. et al. FRA3B extends over a broad region and contains a spontaneous HPV16 integration site: direct evidence for the coincidence of viral integration sites and fragile sites. *Human Molecular Genetics* **5**: 187–95 (1996).

48) Sofos, E. et al. A novel familial 11p15.4 microduplication associated with intellectual disability, dysmorphic features, and obesity with involvement of the ZNF214 gene. *Am J Med Genet A.* 158: 50-8 (2012).

49) Nag, A. et al. Copy number variation at chromosome 5q21.2 is associated with intraocular pressure. *Invest Ophthalmol Vis Sci.* 54: 3607-12 (2013).

50) Filges, I. et al. Familial 14.5 Mb interstitial deletion 13q21.1-13q21.33: clinical and array-CGH study of a benign phenotype in a three-generation family. *Am J Med Genet A*. 149: 237-41 (2009).

51) Tentler, D. et al. A microdeletion in 19q13.2 associated with mental retardation, skeletal malformations, and Diamond-Blackfan anaemia suggests a novel contiguous gene syndrome. *J Med Genet.* 37: 128-31 (2000).

52) Speleman, F. et al. De novo terminal deletion 7p22.1--pter in a child without craniosynostosis. *J Med Genet*. **26**: 528-32 (1989).

53) Shyr, C. et al. FLAGS, frequently mutated genes in public exomes. *BMC Med Genomics.* **7**: 64 (2014).
